# High-temperature adaptation of an *OsNRT2.3* allele is thermoregulated by small RNAs

**DOI:** 10.1126/sciadv.adc9785

**Published:** 2022-11-23

**Authors:** Yong Zhang, Hisae Tateishi-Karimata, Tamaki Endoh, Qiongli Jin, Kexin Li, Xiaoru Fan, Yingjun Ma, Limin Gao, Haiyan Lu, Zhiye Wang, Art E. Cho, Xuefeng Yao, Chunming Liu, Naoki Sugimoto, Shiwei Guo, Xiangdong Fu, Qirong Shen, Guohua Xu, Luis Rafael Herrera-Estrella, Xiaorong Fan

**Affiliations:** ^1^ State Key Laboratory of Crop Genetics and Germplasm Enhancement, College of Resources and Environmental Sciences, Nanjing Agricultural University, Nanjing 210095, China.; ^2^ Key Laboratory of Plant Nutrition and Fertilization in Low-Middle Reaches of the Yangtze River, Ministry of Agriculture, College of Resources and Environmental Sciences, Nanjing Agricultural University, Nanjing 210095, China.; ^3^ Frontier Institute for Biomolecular Engineering Research (FIBER), Konan University, 7-1-20 Minatojima-Minamimachi, Chuo-ku, Kobe 650-0047, Japan.; ^4^ State Key Laboratory of Plant Physiology and Biochemistry, College of Life Sciences, Zhejiang University, Hangzhou, China.; ^5^ Department of Bioinformatics, Korea University, Sejong 30019, Republic of Korea.; ^6^ School of Chemistry and Life Science, Anshan Normal University, Anshan 114007, China.; ^7^ Jiangsu Collaborative Innovation Center for Solid Organic Waste Resource Utilization, College of Resources and Environment Sciences, Nanjing Agricultural University, Nanjing 210095, China.; ^8^ Key Laboratory of Food Quality and Safety of Jiangsu Province, Key Laboratory of Control Technology and Standard for Agro-product Safety and Quality, Institute of Food Safety and Nutrition, Jiangsu Academy of Agricultural Sciences, Nanjing 210014, China.; ^9^ inCerebro Co. Ltd., 8F Nokmyoung Bldg., 8 Teheran-ro10-gil, Gangnam-gu, Seoul 06234, Republic of Korea.; ^10^ Key Laboratory of Plant Molecular Physiology, Institute of Botany, Chinese Academy of Sciences, Beijing 100093, China.; ^11^ Institute of Crop Sciences, Chinese Academy of Agricultural Sciences, Beijing 100093, China.; ^12^ Graduate School of Frontiers of Innovative Research in Science and Technology (FIRST), Konan University, 7-1-20 Minatojima-Minamimachi, Chuo-ku, Kobe 650-0047, Japan.; ^13^ State Key Laboratory of Plant Cell and Chromosome Engineering, Institute of Genetics and Developmental Biology, Innovation Academy for Seed Design, Chinese Academy of Sciences, Beijing 100101, China.; ^14^ Institute of Genomics for Crop Abiotic Stress Tolerance, Department of Plant and Soil Sciences, Texas Tech University, Lubbock, TX 79409, USA.; ^15^ Laboratorio Nacional de Genómica para la Biodiversidad, Unidad de Genómica Avanzada del Centro de Investigación yde Estudios Avanzados del Instituto Politécnico Nacional, 36500 Irapuato, Mexico.

## Abstract

Climate change negatively affects crop yield, which hinders efforts to reach agricultural sustainability and food security. Here, we show that a previously unidentified allele of the nitrate transporter gene *OsNRT2.3* is required to maintain high yield and high nitrogen use efficiency under high temperatures. We demonstrate that this tolerance to high temperatures in rice accessions harboring the HTNE-2 (high temperature resistant and nitrogen efficient-2) alleles from enhanced translation of the *OsNRT2.3b* mRNA isoform and the decreased abundance of a unique small RNA (sNRT2.3-1) derived from the 5′ untranslated region of *OsNRT2.3*. sNRT2.3-1 binds to the *OsNRT2.3a* mRNA in a temperature-dependent manner. Our findings reveal that allelic variation in the 5′ untranslated region of *OsNRT2.3* leads to an increase in OsNRT2.3b protein levels and higher yield during high-temperature stress. Our results also provide a breeding strategy to produce rice varieties with higher grain yield and lower N fertilizer input suitable for a sustainable agriculture that is resilient against climate change.

## Teaser

Temperature-dependent sRNA regulation of the nitrate transporter gene OsNRT2.3 affects nitrogen use efficiency and plant yield.

## INTRODUCTION

Climate change poses a major threat to agricultural systems by reducing global yields of major crops (*1*–*3*). Moreover, the higher expected incidence of drought and heat stress, two consequences of climate change, will severely affect nitrogen (N) uptake and use efficiency, thus further limiting crop production (*4*). In particular, increased nighttime temperatures substantially reduce growth and yield of rice (*Oryza sativa*) (*3*). We compiled phenotypic growth data for 400 rice cultivars from 20 previously published articles and looked for correlations with different growth conditions, as soil chemistry, light irradiation, and temperature profiles experienced by the plants. We calculated the carbon (C) fixation efficiency per N amount (CFEN) value for all varieties as a proxy for biomass production. Our analysis revealed that variation in CFEN among rice varieties is mainly influenced by nighttime temperatures (fig. S1). A clear understanding of the mechanisms by which crops effectively absorb nutrients under increased environmental temperature stress is therefore critical to ensure future food production. Genome-wide association studies and overexpression analysis showed that the high-affinity nitrate transporter *OsNRT2.3* plays a major role in determining N use efficiency (NUE) and yield in rice (*5*–*7*). The *OsNRT2.3* locus produces two mRNA isoforms: *OsNRT2.3a*, with a 42-bp 5′ untranslated region (5′UTR) that accumulates in the root xylem and participates in long-distance nitrate transport from roots to shoots (*8*); and *OsNRT2.3b*, with a 247-bp 5′UTR and lacking a 90-bp intron that is expressed in the phloem of shoots and contributes to nitrate transport from source to sink organs and plays a role in pH and ion homeostasis (*6*). We have previously reported that rice genotypes with higher levels of *OsNRT2.3b* relative to the *OsNRT2.3a* isoform exhibit higher N uptake and transport efficiency (*6*, *9*).

Here, we report on the characterization of rice *OsNRT2.3* haplotypes that have higher agronomic nitrogen use efficiency (ANUE; contribution of unit nitrogen fertilizer application to increase rice yield) and yield at higher nighttime temperatures. We found that a small RNA (sRNA; sNRT2.3-1) regulates *OsNRT2.3a* and *OsNRT2.3b* levels in a temperature-dependent manner. Our results uncovered a complex mechanism of regulation of *OsNRT2.3* that has a great impact on ANUE and yield, opening new directions for the molecular breeding of rice varieties with higher NUE and yield at elevated temperatures.

## RESULTS AND DISCUSSION

### A single-nucleotide polymorphism of *OsNRT2.3* confers high-temperature adaptation for sustainable yield improvement in rice

**Fig. 1.**
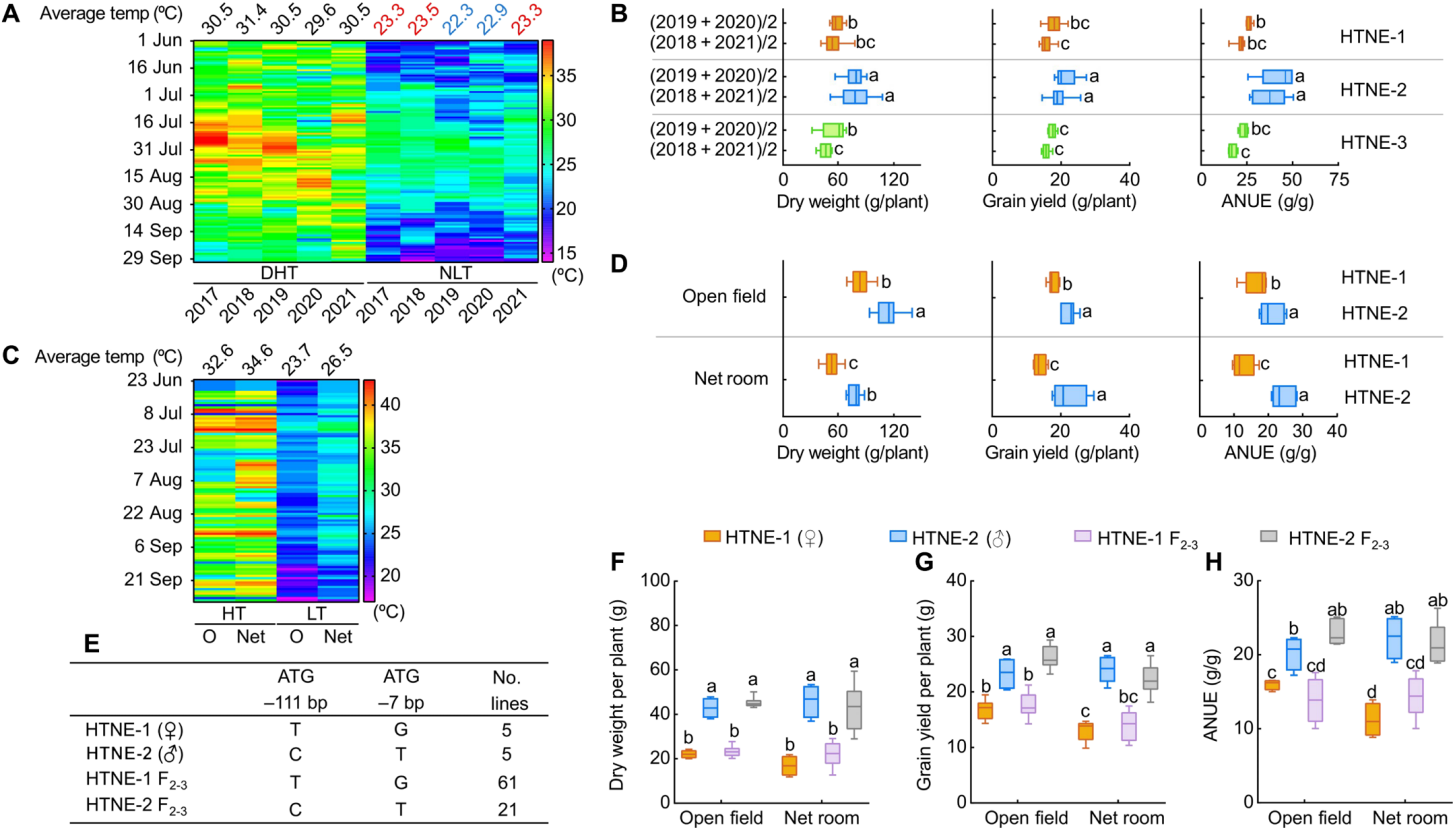
Phenotype of HTNE-1 and HTNE-2 accessions grown at different temperatures. Plants were grown under 60 kg N/ha (low N) or 120 kg N/ha (high N). (**A**) High- and low-temperature profiles from 1 June to 30 September every year from 2017 to 2021 in Lishui District, Nanjing, shown as a heatmap. DHT, day high temperature; NLT, night low temperature. (**B**) Average dry weight, grain yield, and ANUE for rice accessions carrying the HTNE-1, HTNE-2, or HTNE-3 allele for 2 years with similar NLTs: 2019 and 2020 as well as 2018 and 2021. The accessions with the HTNE-1 allele have no SNPs in *OsNRT2.3*; accessions with the HTNE-2 allele have SNPs at position −111 upstream of the *OsNRT2.3* ATG (T to C) and at position −7 (G to T); accessions with the HTNE-3 allele harbor an insertion and other SNPs in *OsNRT2.3*. (**C**) High- and low-temperature profiles of the open field or the net room from 23 June to 30 September 2021. OHT, open-field high temperature; OLT, open-field low temperature; NetHT, net room high temperature; NetLT, net room low temperature. (**D**) Dry weight, grain yield, and ANUE of HTNE-1 and HTNE-2 accessions grown in the open field and net room. Error bars, SE (*n* ≥ 5 biological replicates). (**E**) SNPs in recombinant plants from the BC_1_F_2_ population derived from a cross between Xindao 34 (HTNE-1) and Ka821asq (HTNE-2). (**F** to **H**) Dry weight (F), grain yield (G), and ANUE (H) of HTNE-1, HTNE-2, and BC_1_F_2-3_ plants grown in the open field or the net room. HTNE-1 (♀), mother line; HTNE-2 (♂), father line; (*n* = 5 biological replicates). HTNE-1^TG^, from BC_1_F_2_ with HTNE-1–type polymorphisms (*n* = 61 biological replicates). HTNE-2^CT^, from BC_1_F_2_ with HTNE-2–type polymorphisms (*n* = 21 biological replicates). Different lowercase letters indicate significant differences (*P* < 0.05; unpaired two-tailed Student’s *t* test).

From 2017 to 2021, we evaluated 239 rice accessions (*indica*, *japonica*, and Aus species), collected in different regions of several countries (China, Japan, South Korea, the Philippines, Thailand, Bangladesh, Kenya, India, the United States, and Italy) for their performance when grown in the field or in net rooms and experiencing natural seasonal variation in temperatures. We observed significant differences in their ability to adapt to high nighttime temperatures, as reflected by yield and NUE values (Fig. 1, A to D, and fig. S2, A to C). We determined that the *OsNRT2.3* locus exists as three main alleles among the 239 rice accessions, designated here as high temperature resistant and nitrogen efficient 1 {HTNE-1; no single-nucleotide polymorphisms [SNPs] relative to the reference genome of Nipponbare [Nip; *O. sativa* L. spp. *japonica*], HTNE-2 [with two linked SNPs, 111 bp (T or C) and 7 bp (G or T) upstream of the *OsNRT2.3* initiation codon (ATG)], and HTNE-3 [SNPs in the *OsNRT2.3* promoter]} (fig. S2D). Here, we report that rice accessions harboring the HTNE-2 haplotype have higher growth, yield, and NUE when grown in the field during years with high night temperatures and in net rooms in which the nighttime temperature was set 1°C higher than under open-field conditions (Fig. 1, A to D, and figs. S2 to S5). To ascertain that the observed greater tolerance to high nighttime temperature is associated with the HTNE-2 haplotype, we crossed the rice accessions Xindao 34 (HTNE-1 haplotype) and Ka821asq (HTNE-2 haplotype) and selected recombinant plants from the BC_1_F_2_ generation homozygous for each haplotype. We then used their F_3_ offspring (BC_1_F_2-3_) with the HTNE-1 or HTNE-2 haplotype to determine NUE in the open field and in net rooms with an application of 60 kg N/ha (low N) or 120 kg N/ha (high N). We observed that the lines carrying HTNE-2 perform better for all growth metrics measured and had higher yield and NUE in both the field and the net room (Fig. 1, E to H, and table S1). The results together provided genetic evidence linking HTNE-2 with high rice yield and NUE when exposed to high night temperature.

**Fig. 2.**
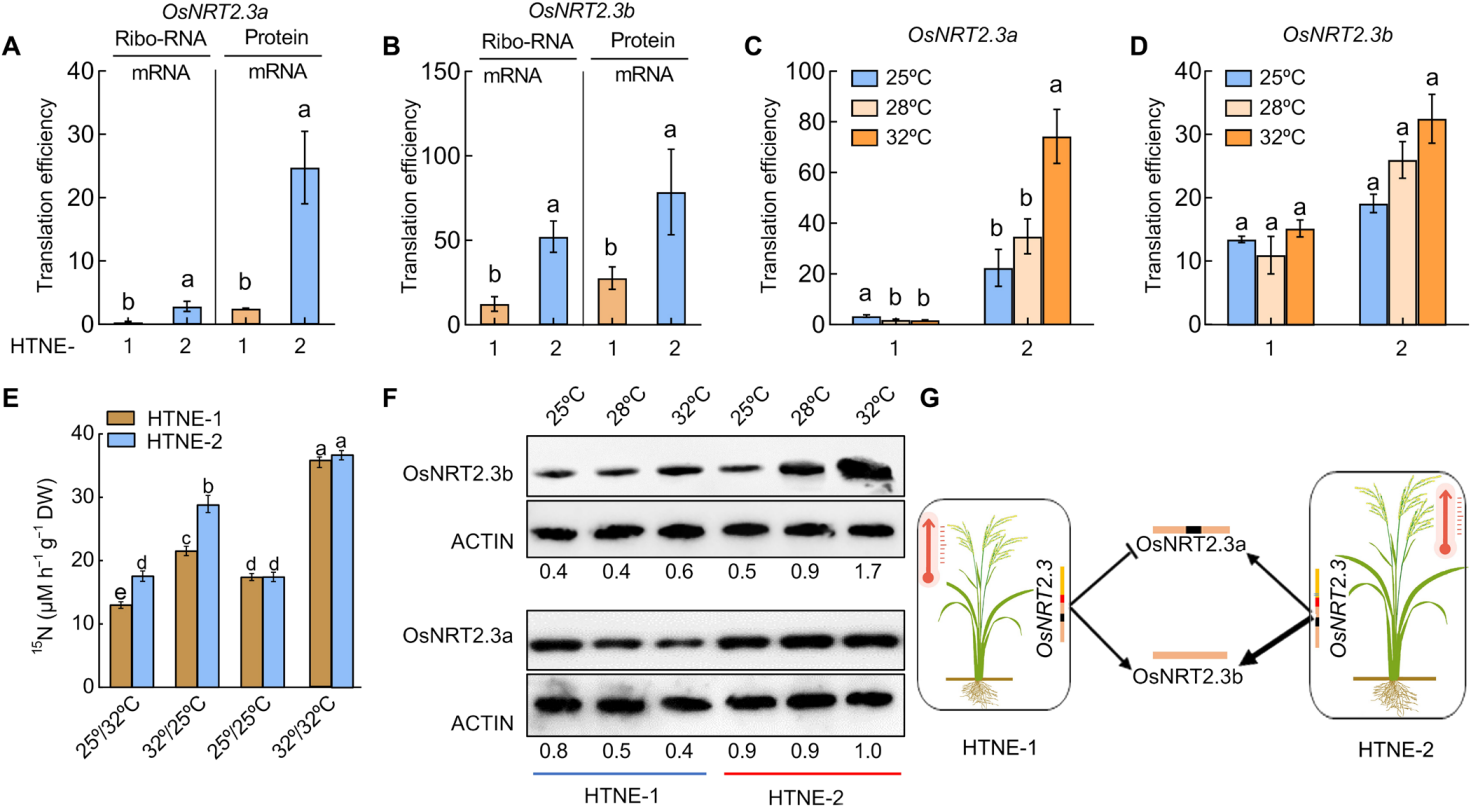
Effects of OsNRT2.3a and OsNRT2.3b protein lifetime on nitrogen uptake. Translation efficiency of *OsNRT2.3a* (**A**) and *OsNRT2.3b* (**B**) in HTNE-1 and HTNE-2 plants. Ribo-RNA/mRNA is the ratio between the abundance of ribosomal RNA and that of mRNA. Protein/mRNA is the ratio between protein abundance and mRNA under field conditions at the booting stage. (**C** and **D**) Translation efficiency of *OsNRT2.3a* (C) and *OsNRT2.3b* (D) mRNA in HTNE-1 and HTNE-2 plants grown under different temperatures (25°, 28°, or 32°C in a growth chamber at the seedling stage). (**E**) ^15^N uptake in HTNE-1 and HTNE-2 plants grown under different temperature regimes. ^15^NO_3_^−^ was provided as a 0.1 mM Ca(NO_3_)_2_ solution. Four temperature treatments (night/day) were tested: 25°/32°C (control treatment), 32°/25°C, 25°/25°C, and 32°/32°C. DW, Dry weight. Data are shown as means ± SE (*n* = 3 biological replicates). (**F**) OsNRT2.3a and OsNRT2.3b protein abundance in HTNE-1 and HTNE-2 plants grown at different temperatures (25°, 28°, or 32°C), as determined by immunoblotting. Relative band intensities were determined with ImageJ software (National Institutes of Health) and are given below the immunoblots. (**G**) The simple structure diagram of two *OsNRT2.3* alternative spliced proteins, OsNRT2.3a and OsNRT2.3b, under high nighttime. The thickness of the line represents the strength. Different lowercase letters indicate significant differences (*P* < 0.05; unpaired two-tailed Student’s *t* test).

To investigate the possible mechanism by which HTNE-2 plants are more tolerant to high nighttime temperatures, we examined relative *OsNRT2.3* transcript levels in the different haplotypes. We observed that the level for the *OsNRT2.3b* transcript isoform is higher and those of the *OsNRT2.3a* isoform lower in accessions with the HTNE-2 haplotype than in accessions with the HTNE-1 or HTNE-3 haplotype (fig. S6, A and B). High temperature (32°C) decreased the abundance of the *OsNRT2.3a* isoform in both HTNE-1 and HTNE-2 plants and resulted in lower *OsNRT2.3b* transcript levels in HTNE-1 but not in HTNE-2 plants (fig. S6, C and D), suggesting that the SNPs located in the *OsNRT2.3* 5′UTR of HTNE-2 have a differential influence on the transcription of the two *OsNRT2.3* mRNA isoforms in a temperature-dependent manner. In addition, we discovered that *OsNRT2.3b* is translated 3 to 31 times more efficiently than *OsNRT2.3a* in all rice accessions grown in the field and sampled at the booting stage; notably, HTNE-2 accessions also exhibited a three to nine times higher translation efficiency for both *OsNRT2.3* mRNA isoforms relative to HTNE-1 accessions (Fig. 2, A and B, and fig. S6, E and F). HTNE-2 plants also displayed higher N uptake and accumulation compared to HTNE-1 plants under high nighttime temperatures but not at constant high or low temperature (Fig. 2E and fig. S7). Moreover, higher temperature substantially decreased OsNRT2.3a protein abundance and mRNA translation efficiency in HTNE-1 but not in HTNE-2 plants (Fig. 2, C, D, F, and G, and table S2). This observation suggested that OsNRT2.3a and OsNRT2.3b proteins are more abundantly expressed in HTNE-2 plants at high temperature than in HTNE-1 plants.

**Fig. 3.**
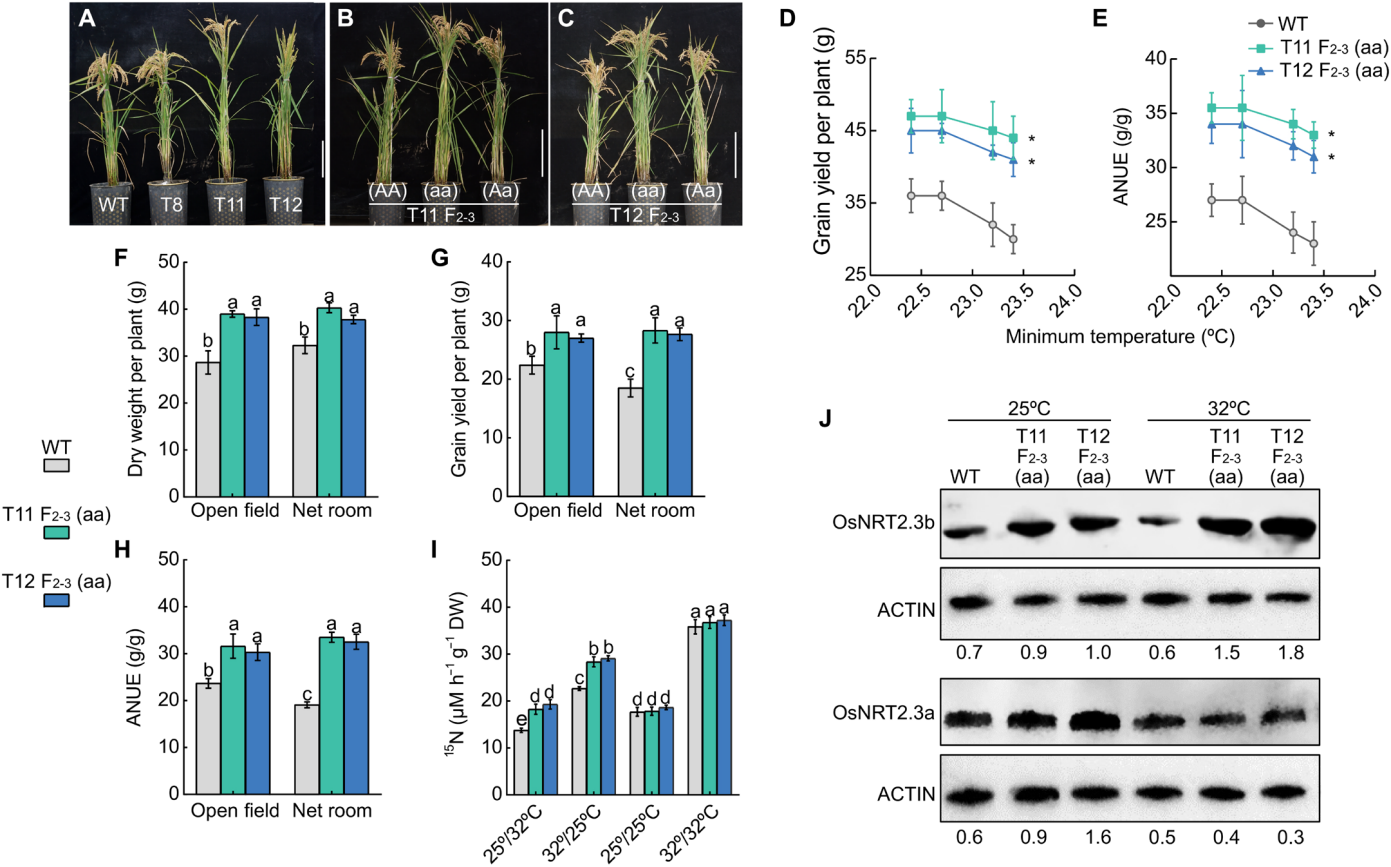
A mutation 83 bp upstream of the *OsNRT2.3* ATG enhances adaptation to high temperatures. (**A**) Characterization of *OsNRT2.3* tilling lines (T8, T11, and T12). Gross morphology of the BC_1_F_3_ generation for T11 backcross lines (**B**) and T12 backcross lines (**C**). Grain yield (**D**) and ANUE (**E**) of *OsNRT2.3* tilling lines [T11 F_2-3_ (aa) and T12 F_2-3_ (aa)] and the wild type (WT; ZH11 and HTNE-1 allele) when grown under different temperatures in 2017, 2018, 2019, and 2020 at Nanjing, China. The asterisks indicate statistically significant differences relative to the WT [*P* < 0.05; unpaired two-way analysis of variance (ANOVA)]. Dry weight (**F**), grain yield (**G**), and ANUE (**H**) of *OsNRT2.3* tilling lines and the WT grown in the open field or a net room. Data are shown as means ± SE (*n* = 5 biological replicates). (**I**) ^15^N uptake in *OsNRT2.3* tilling lines and the WT exposed to different temperature regimes. ^15^NO_3_^−^ was supplied as a 0.1 mM Ca(NO_3_)_2_ solution. Data are shown as means ± SE (*n* = 5 biological replicates). Different lowercase letters indicate significant differences between *OsNRT2.3* tilling lines and WT (*P* < 0.05; unpaired two-tailed Student’s *t* test). (**J**) OsNRT2.3a and OsNRT2.3b protein abundance in *OsNRT2.3* tilling lines and the WT grown at 25° or 32°C, as determined by immunoblot. Relative band intensities were determined with ImageJ software and are given below the immunoblots.

To obtain additional evidence that mutations in the *OsNRT2.3* 5′UTR affect the expression levels of both mRNA isoforms and result in higher yield and NUE under high nighttime temperatures, we searched for tilling mutants in the *OsNRT2.3* 5′UTR. We identified three lines with a single T-to-C mutation 83 bp upstream of the *OsNRT2.*3 ATG (Fig. 3A and fig. S8A). To assess the contribution of this mutation to yield and NUE, we backcrossed the *OsNRT2.3* tilling lines T11 and T12 to their wild-type (WT) Zhonghua 11 (ZH11) and selected BC_1_F_2_ plants homozygous for the WT or mutant allele (Fig. 3, B and C). We observed higher rice growth, yield, and NUE under high nighttime temperature conditions in the BC_1_F_2-3_ progeny harboring the mutation but not those with the WT allele (Fig. 3, D to H, and figs. S8 and S9). As with HTNE-2 plants, *OsNRT2.3* tilling lines had increased nitrate uptake and accumulation under high nighttime temperature conditions but not under constant high and low temperatures (Fig. 3I and fig. S10). Also, as in HTNE-2 plants, *OsNRT2.3b* transcripts and OsNRT2.3b protein accumulated to higher levels in the *OsNRT2.3* tilling lines than in their WT siblings, with OsNRT2.3b protein abundance increasing in temperatures from 25° to 32°C (Figs. 2F and 3J and table S2). Quantitative reverse transcription polymerase chain reaction (qRT-PCR) expression analysis of stable transgenic lines confirmed that a version of the *OsNRT2.3* promoter with the T-to-C mutation, present in the tilling lines, directs higher transcript levels of *OsNRT2.3b* while reduced levels of *OsNRT2.3a* when compared to the intact *OsNRT2.3* promoter (fig. S11). The finding that yield and NUE in *OsNRT2.3* tilling lines are similar to those obtained for HTNE-2 plants confirmed a tight link between higher *OsNRT2.3b* protein abundance and the ability to maintain high yield and NUE when exposed to high-temperature stress (Figs. 1, 2, F and G, and 3, D to J, and table S2).

### sRNAs promote the adaptation of rice to high temperature by regulating the transcription of *OsNRT2.3*

**Fig. 4.**
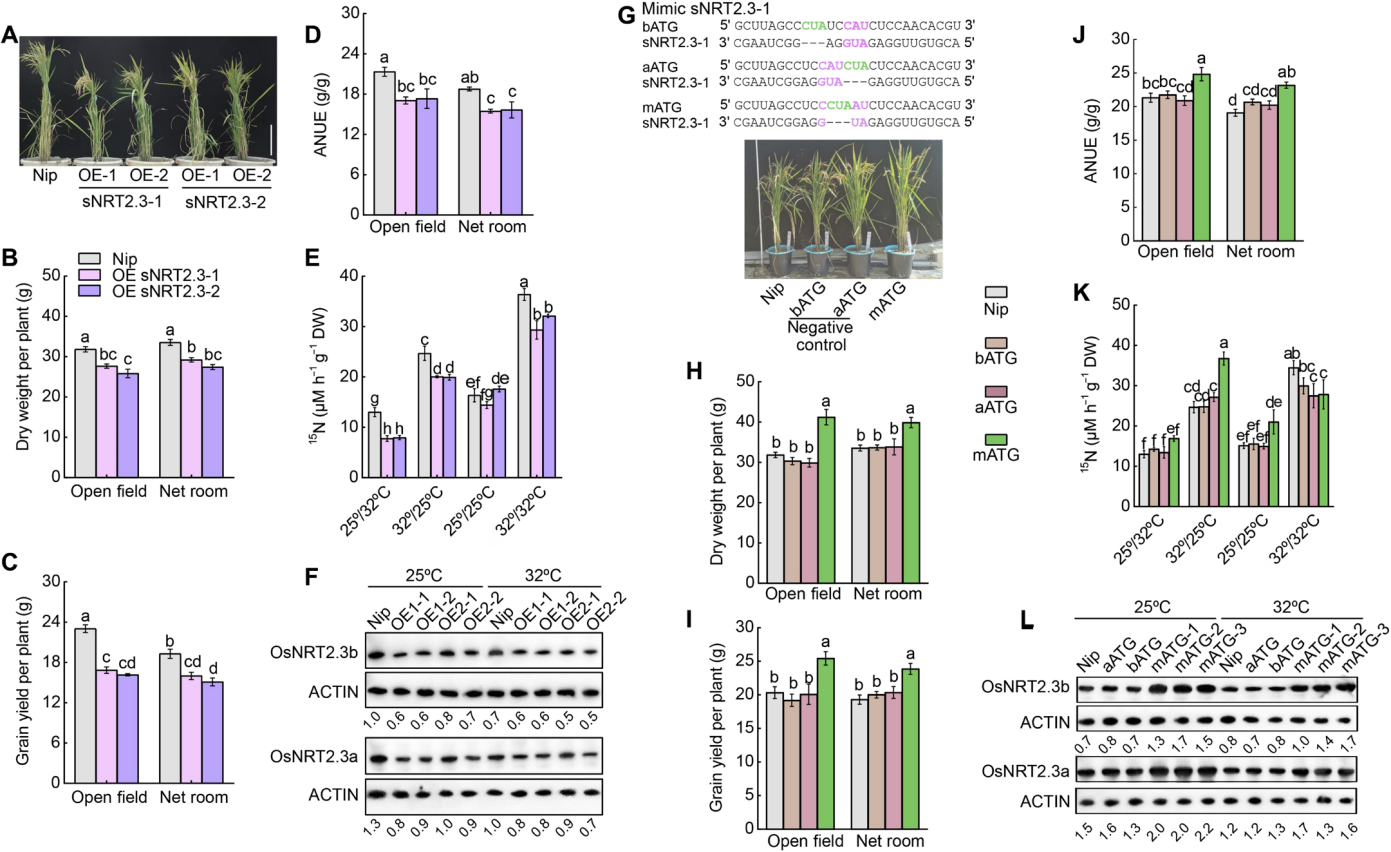
sRNAs alter the adaptation of rice to high temperature. (**A**) Morphology of sNRT2.3-1– and sNRT2.3-2–overexpressing lines in Nip. (**B** to **D**) Dry weight (B), grain yield (C), and ANUE (D) of the sNRT2.3-1– and sNRT2.3-2–overexpressing lines and Nip grown in the open field and in a net room. (**E**) ^15^N uptake in the sNRT2.3-1– and sNRT2.3-2–overexpressing lines and Nip subjected to different temperature regimes. ^15^NO_3_^−^ was supplied as a 0.1 mM Ca(NO_3_)_2_ solution. (**F**) OsNRT2.3a and OsNRT2.3b protein abundance in the sNRT2.3-1 and sNRT2.3-2 overexpression lines grown at 25° or 32°C, as determined by immunoblotting. (**G**) Characterization of sNRT2.3-1 mimic lines in Nip. (**H** to **J**) Dry weight (H), grain yield (I), and ANUE (J) of the sNRT2.3-1 mimic lines and WT-N grown in the open field and in a net room. (**K**) ^15^N uptake in the sNRT2.3-1 mimic lines and WT-N subjected to different temperature regimes. ^15^NO_3_^−^ was supplied as a 0.1 mM Ca(NO_3_)_2_ solution. (**L**) OsNRT2.3a and OsNRT2.3b protein abundance in the sNRT2.3-1 mimic lines, as determined by immunoblotting. Data are shown as means ± SE (*n* = 5 biological replicates). Different lowercase letters indicate significant differences between the lines and Nip (*P* < 0.05; unpaired two-tailed Student’s *t* test).

To understand how the expression of *OsNRT2.3a/b* is regulated by temperature in rice plant, we sequenced the RNA of *OsNRT2.3a* and *OsNRT2.3b* under different temperatures, and we found that there are two unexpected short RNA transcripts that overlap with the *OsNRT2.3*a/b genic region. Reports have shown that sRNAs participate in the regulation of gene expression and translation (*10*–*12*). Therefore, we searched for sRNAs that might target *OsNRT2.3* transcripts, leading to the identification of two 24-nucleotide (nt) sRNAs mapping to the 5′ region of *OsNRT2.3*, named here sNRT2.3-1 and sNRT2.3-2 (fig. S12, A and B). Both sRNAs were mainly expressed in the shoots and to lower levels in the root of all genotypes tested (fig. S12, C and D). Under field conditions at the booting stage, the abundance of sNRT2.3-1 and sNRT2.3-2 was lower in HTNE-2 plants and *OsNRT2.3* tilling lines than in HTNE-1 plants (fig. S12, E to H). In addition, we noticed that the levels of sNRT2.3-1 and sNRT2.3-2 are also regulated by temperature and increased at 32°C compared to 25°C (fig. S12I). Overexpression of either sRNA diminished rice growth, yield, and NUE under open-field and net room conditions (Fig. 4, A to D, and figs. S13 and S14). Overexpression of sNRT2.3-1 or sNRT2.3-2 had a negative effect on N uptake and accumulation under high nighttime temperatures and under constant high temperature but had no effect when overexpression lines were grown at a constant low temperature (Fig. 4E and fig. S15). Analysis of sRNA overexpression rice lines showed that sNRT2.3-1 decreases the transcription and/or the translation of both *OsNRT2.3a* and *OsNRT2.3b* mRNA isoforms, while sNRT2.3-2 overexpression mainly affected *OsNRT2.3b* (Fig. 4F; fig. S13, C and D; and table S2). sRNAs alter the adaptation of rice to high nighttime temperature by reducing the accumulation of OsNRT2.3 protein.

To confirm the role of sNTR2.3-1 on *OsNRT2.3* transcription, we used a target mimicry strategy using complementary mutated versions of sNTR2.3-1 to sequester this sRNA and prevent binding to its target mRNA (Fig. 4G and fig. S16). Accordingly, we generated three sets of transgenic lines: the after ATG and before ATG lines, in which we inserted a CUA (mismatch loop) sequence after (aATG) or before (bATG) the CAU (the reverse complementary sequence of the AUG at the center of the sNTR2.3-1/*OsNRT2.3* duplex); and the middle ATG (mATG) lines, in which a CUA was inserted within the CAU sequence of sNRT2.3-1 (Fig. 4G). The mATG lines exhibited increased growth, yield, and NUE when grown in the open field and net room (Fig. 4, G to J, and fig. S17). The mATG lines also displayed increased N uptake and accumulation under high night temperature and constant low temperature but not under sustained high temperature (Fig. 4K and fig. S18). Moreover, we established that the mATG lines had substantially increased levels of both *OsNRT2.3a* and *OsNRT2.3b* transcripts and OsNRT2.3b protein but only had a slight effect on OsNRT2.3a (Fig. 4L and fig. S16). Field data demonstrated that the mATG lines have higher yield and N accumulation compared to their WT Nip under various levels of N fertilizer supply, suggesting that the reduced level of active sNRT2.3-1 has a similar effect as that caused by the SNPs present in HTNE-2 plants (fig. S19).

### The structure of sRNA, *OsNRT2.3a/b* transcription, and translation efficiency are sensitive to temperature shift from 25° to 32°C

**Fig. 5.**
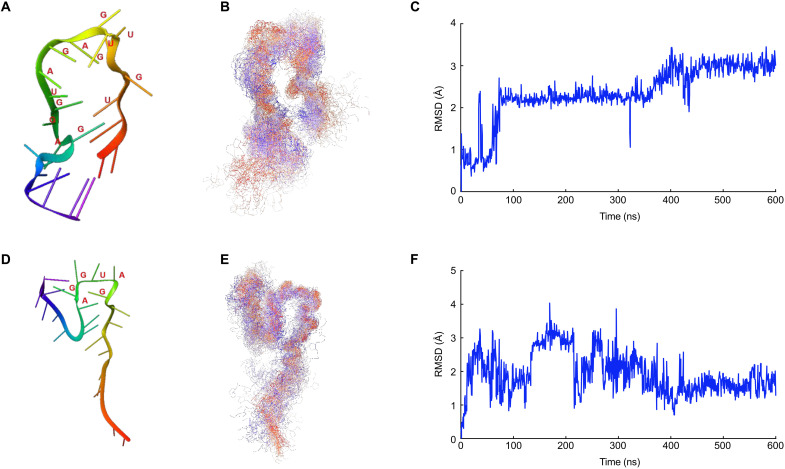
Effects of temperature on the tertiary structure of sRNA hairpin folding. (**A**) Average sNRT2.3-1 structures at 25°C, extracted by sampling 101 structures obtained from 600-ns sRNA MD simulations. The sequence UGUUGGAGAUGGAG folded into a hairpin at 25°C. (**B**) Extracted and all 101 sampled sNRT2.3-1 structures from a 600-ns MD simulation at 25°C, aligned to relative the 101 sRNA backbones. (**C**) Root mean square deviation (RMSD) values of sNRT2.3-1 hairpin at 25°C between nucleotides U4 and G17 of sRNA2.3-1 (UCGUGUUGGAGAUGGAGGCUAAGC) over a 600-ns MD simulation. (**D**) Average sNRT2.3-1 structures at 32°C, extracted by sampling 101 structures obtained from 600-ns sRNA MD simulations. The sequence GAUGGA folded into a short hairpin at 32°C. (**E**) Extracted and all 101 sampled sNRT2.3-1 structures from a 600-ns MD simulation at 32°C, aligned relative to the 101 sRNA backbones. (**F**) RMSD values of sNRT2.3-1 at 32°C between nucleotides G11 and A16 of sNRT2.3-1 (UCGUGUUGGAGAUGGAGGCUAAGC) over a 600-ns MD simulation.

sRNA folding is strongly temperature dependent (*13*). Subtle changes can occur in overall RNA structure with varying temperature. We conducted molecular dynamic (MD) simulations to test whether temperature might alter the three-dimensional (3D) structure of sNRT2.3-1 and sNRT2.3-2. We determined that an increase from 25° to 32°C altered the hairpin loop structure of sNRT2.3-1 (Fig. 5). Under the same settings, MD simulations for sNRT2.3-2 indicated that the structure of sNRT2.3-2 is highly flexible at 25°C (fig. S20, A and B), whereas at 32°C, sNRT2.3-2 structure appeared steady for the first 300 ns before becoming unstable, but it stabilized again after 400 ns (fig. S20, C and D). For alignment analysis, we recovered the averaged structures of sNRT2.3-2 for the 0 to 300–ns and 400 to 600–ns periods, although the shape of the two averaged structures differed (fig. S20, C and D). Notably, there were no substantial changes in the structure of the GAGUUCUGGCUC hairpin loops (sNRT2.3-2 partial sequence) during the 0 to 300–ns and 400 to 600–ns periods at 32°C (fig. S20, D and E). As for sNRT2.3-1, the temperature shifts from 25° to 32°C changed the hairpin loop sequences from UGUUGGAGAUGGAG into GAUGGA (Fig. 5, A and D), which would affect the binding to the target mRNA (*14*, *15*).

**Fig. 6.**
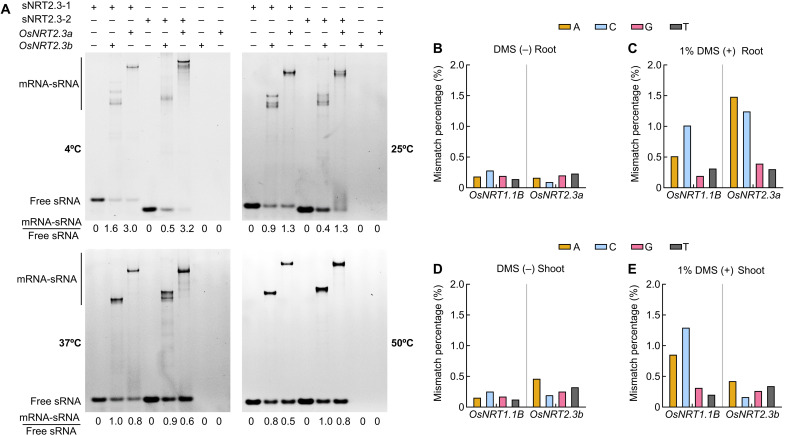
Effect of temperature and presence of an *OsNRT2.3b* intron on the secondary structure of *OsNRT2.3a* and *OsNRT2.3b* mRNAs. (**A**) In vitro binding of sNRT2.3-1 and sNRT2.3-2 to *OsNRT2.3a* and *OsNRT2.3b* mRNAs at 4°, 25°, 37°, and 50°C. sNRT2.3-1 and sNRT2.3-2 were labeled with 6-Carboxyfluorescein (6-FAM) at the 5′ end. Gels were imaged for 6-FAM fluorescence of sNRT2.3-1 and sNRT2.3-2. mRNA-sRNA/free sRNA, the ratio of mRNA-sRNA complex to free sRNA. (**B** to **E**) Nip seedlings were treated with 1% (v/v) DMS or not treated with DMS as control. Seedlings were split into roots and shoots and assessed separately. Total mismatch percentage for each nucleotide of two genes, *OsNRT1.1B* and *OsNRT2.3a*, in DMS-untreated (B) and DMS-treated (C) root samples. *OsNRT1.1B* served as the control. Total mismatch percentage for each nucleotide of two genes, *OsNRT1.1B* and *OsNRT2.3b*, in DMS-untreated (D) and DMS-treated (E) shoot samples.

Temperature has a crucial effect on sRNA folding, and changes in RNA conformations can regulate their function (*16*). Furthermore, long RNAs, like mRNAs, can also form several topologies, and their structures may change with temperature. RNA folding also determines the size and shape of the binding site of sRNA. Under high-temperature conditions, sNRT2.3-1 accumulated to high levels and was more likely to interact with the *OsNRT2.3a* mRNA isoform than *OsNRT2.3b* (Fig. 6A and figs. S6, C and D, S12I, and S21). The binding of sNRT2.3-1 to the *OsNRT2.3a* mRNA isoform could further promote its degradation and inhibit its translation (Figs. 2F, 3J, and 4, F and L; figs. S13C and S16A; and table S2). To test for changes in sRNA binding, we used dimethyl sulfate (DMS) modification of base-pairing faces for structural analysis of RNAs. Mapping DMS activity sites showed that *OsNRT2.3a* mRNA reacts more strongly with DMS than *OsNRT2.3b* did (Fig. 6, B to E), suggesting that *OsNRT2.3b* transcripts may have a stronger RNA secondary structure or be tightly bound by protein complexes in vivo. Plants typically have a single binding site for sRNA-mRNA interactions (*17*). Changing the temperature affected the conformation of sNRT2.3-1 as well as that of its binding site (Figs. 5 and 6A and fig. S21). We observed that sNRT2.3-1/2 have two different binding sites in *OsNRT2.3b* at lower temperature (Fig. 6A and fig. S21). In addition, because of the difference in affinity between mRNA and sRNA, sRNA binding at high temperatures is generally weaker than at low temperatures. The lower levels of sNRT2.3-1/2 in HTNE-2 plants were accompanied by a lower inhibition of *OsNRT2.3b* translation, thereby promoting higher translation efficiency and OsNRT2.3b protein abundance (Figs. 2, B and F, and 3J; figs. S6 and S12, E to H; and table S2). These results support the effect of sRNA binding to *OsNRT2.3a*, accelerating its degradation in a temperature-dependent manner.

**Fig. 7.**
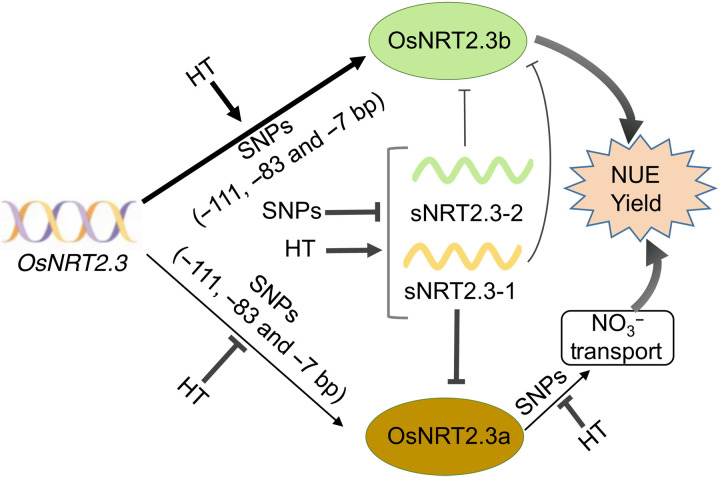
The schematic diagram of the mechanism by which *OsNRT2.3* regulates NUE and yield at nighttime high temperature. SNPs, the SNPs that appeared in plants of HTNE-2 [−111 upstream of the *OsNRT2.3* ATG (T to C) and at position −7 (G to T)] and *OsNRT2.3* tilling lines [−83 bp upstream of the *OsNRT2.3* ATG (T to C)]. HT, high nighttime temperature. Circles, proteins of OsNRT2.3a and OsNRT2.3b. The thickness of the line represents the strength.

In summary, *OsNRT2.3a* is predominantly expressed in root xylem and is responsible for root-to-shoot N transport (*6*, *8*). Although knockdown of *OsNRT2.3a* by RNA interference decreases root-to-shoot N transport and shoot N accumulation and thus results in reduced plant growth, overexpression of *OsNRT2.3a* does not increase N uptake or NUE in rice plants (*6*, *8*, *9*). *OsNRT2.3b* is expressed in phloem cells in shoots and transports N from source to sink organs, and its overexpression increases N uptake and NUE (*6*). Furthermore, overexpression of *OsNRT2.3b* increases iron and phosphorus transport to leaves and reduces photorespiration, thus increasing biomass and yield due to its pH balancing function in phloem cells (*6*, *7*). The temperature experienced by rice roots in paddy soils is around 25°C, while shoots are exposed to a temperature of about 30°C 1 to 2 cm above the water surface during daytime (*18*). Leaf temperature in the summer can reach 25° to 30°C at night and 30° to 40°C at daytime (*19*); therefore, local temperature differentially affects the expression of *OsNRT2.3a* and *OsNRT2.3b* (fig. S6, C and D). Higher temperatures do not affect the transcript levels of *OsNRT2.3b* in HTNE-2 plants, as they do in HTNE-1 plants (fig. S6, C and D), thus explaining, at least in part, why HTNE-2 plants maintain higher NUE and yield in years with higher nighttime temperatures (Figs. 1 and 7 and figs. S4 and S5). Furthermore, we determined that the abundance of the sRNA sNRT2.3-1 is higher in HTNE-1 plants than HTNE-2 plants and tilling lines (fig. S12, E and G). The interaction between sNRT2.3-1 and *OsNRT2.3a* but not with *OsNRT2.3b* explains the lower translation efficiency of *OsNRT2.3a* than *OsNRT2.3b* in HTNE-1 plants (Fig. 2, A and B). As temperature increases sNRT2.3-1 abundance in HTNE-1 plants, the effect of sNRT2.3-1 on *OsNRT2.3a* is more pronounced in HTNE-1 plants than in HTNE-2 plants and tilling lines in which sNRT2.3-1 accumulates to much lower levels and thus has a lower effect on OsNRT2.3a translation (Figs. 2, F and G, 3J, and 7; fig. S12, E to I; and table S2). In the case of *OsNRT2.3a*, knockdown lines accumulate less N and show reduced plant growth (*8*). We established that HTNE-1 plants have lower levels of *OsNRT2.3a* than HTNE-2 plants and that higher temperatures decrease the translation efficiency of *OsNRT2.3a* mRNA in HTNE-1 but not HTNE-2 plants (Fig. 2, C and D). Furthermore, these differences in expression are due to SNPs that, in HTNE-2 and tilling lines, change in the binding sites and binding efficiency of sRNA to mRNA, resulting from structural changes in mRNA and sRNA. Together, these results suggest that HTNE-2 plants and tilling lines perform better during years of high nighttime temperature because of their higher levels of *OsNRT2.3a* and *OsNRT2.3b*, which are less affected by high nighttime temperature (Fig. 7). The polymorphisms identified in the HTNE-2 haplotype, in addition to loss of sNRT2.3-1 function, offer new targets for crop breeding with greater tolerance to high temperature and high yield.

## MATERIALS AND METHODS

### Multivariate regression analysis for CFEN

To determine the factors influencing CFEN in different rice varieties, a multivariate regression tree analysis was conducted. The data for aboveground nitrogen contents, biomass, and yield of rice were extracted from 20 peer-reviewed articles published between 1995 and 2012, with a total sample size of 400. Other relevant information such as soil types, management methods, nitrogen supply, rice varieties, and countries was also included in the analysis. The influencing factors of CFEN were divided into several categories: rice variety (*indica* rice, *japonica* rice, conventional rice, and hybrid rice), the amount of nitrogen supply, ratio of base fertilizer, and country.

Meteorological parameters were collected on the basis of the longitude and altitude of each test site. These meteorological parameters included average temperature, average amount of light irradiation, and average photoperiod during the rice growing season. The multivariate regression tree analysis was then constructed using the mvpart function of the mvpart package in R, with different factors such as rice variety, nitrogen supply, ratio of base fertilizer, country, longitude, latitude, temperature, daylight hours, and insolation. SPSS16.0 and Microsoft Excel 2007 were used for statistical analysis. Multiple comparisons of all the data were carried out using the least significant difference test. The formula for calculating CFEN was as follows:

CFEN = carbon contents in aboveground organ/nitrogen contents in aboveground organ.

### Plant materials and growth conditions

Natural varieties were planted at the experimental base of Baima Academy of Agricultural Sciences, Lishui District, Nanjing, in the middle and lower reaches of the Yangtze River. Fertilization in the experimental field consisted of total N content of 240 kg N/ha for 2017 and 120 kg N/ha (high nitrogen) and 60 kg N/ha (low nitrogen) for 2018 to 2021, P_2_O_5_ content of 60 kg/ha, and K_2_O content of 150 kg/ha.

*OsNRT2.3* tilling and transgenic lines for field experiments were grown in fields specifically authorized for handling transgenic rice in Baguazhou, Nanjing. The chemical properties of the soils shown in the plots were described by Chen *et al*. (*20*).

The BC_1_F_2-3_ population derived from a cross between HTNE-1 (Xindao 34, ♀) and HTNE-2 (Ka821asq, ♂) was planted in the open field and in a net room at the experimental base of Baima Academy of Agricultural Sciences. Fertilization in the open field and net room consisted of total N contents of 120 kg N/ha (high nitrogen) and 60 kg N/ha (low nitrogen), P_2_O_5_ content of 60 kg/ha, and K_2_O content of 150 kg/ha. The foliar temperature of rice grown in the open field and net room was monitored in real time from 23 June to 30 September 2021 using 4G temperature sensors to compare night and day temperatures (shown in Fig. 1C). Genomic DNA was extracted from individuals of the BC_1_F_2_ population, sequenced, and compared to the parental genome sequence of *OsNRT2.3*. The sequences of PCR primers are given in table S3.

For hydroponic growth conditions, rice at three leaves and one heart stage was grown in a temperature-controlled incubator and treated with 0.1 mM Ca(NO_3_)_2_ and different temperatures for 1 week. Four temperature conditions (night/day) were tested: 25°/32°C (control treatment), 32°/25°C, 25°/25°C, and 32°/32°C. The dry weight and nitrogen contents were determined for roots and shoots. The expression of *OsHSP70*, encoding heat shock protein 70, was selected to assess the efficacy of the temperature treatments, with *OsActin* and *OsTubulin* serving as reference genes (fig. S22). The expression levels of *OsHSP70* at different temperature were in line with a previous report (*21*).

### Determination of total nitrogen contents

Samples were harvested and heated at 105°C for 30 min and ground into powder. The method of H_2_SO_4_-H_2_O_2_ digestion was used. Total nitrogen concentration in rice was measured by the Kjeldahl method on a continuous flow analyzer (SEAL AutoAnalyzer 3).

N harvest index (%) was calculated as (grain N accumulation at maturity/total N accumulation at maturity) × 100; ANUE (g/g) was calculated as (grain yield per unit area in the region with high N rate grain yield per unit area in the region of lower N rate)/difference in N application rate per unit area between two regions. Physiological NUE (g/g) was calculated as grain yield per plant/total N content of aboveground organ per plant.

### Temperature data collection

Maximum and minimum temperatures from 1 June to 30 September 2017 to 2021 were collected from the website of the China weather bureau.

### Immunoblot analysis

Antibodies specifically recognizing OsNRT2.3a or OsNRT2.3b were generated as described in (*6*, *8*). Total protein was extracted from the backcross lines carrying the mutation at position −83 bp, as well as from plants harboring the HTNE-1 and HTNE-2 haplotypes for SDS–polyacrylamide gel electrophoresis. Proteins were then transferred to polyvinylidene difluoride (PVDF) membranes, and the membranes were incubated with antibodies against OsHSP (China, catalog no. AbP80236-A-SE) or OsACTIN (Sigma-Aldrich, catalog no. A0480) (1:5000) and OsNRT2.3a (1:500) or OsNRT2.3b (1:500) overnight at 4°C with 5% Tris-buffered saline with Tween®20 detergent (TBST) buffer. The PVDF membrane was then incubated with the rabbit secondary antibody (1:1000; Sigma-Aldrich), followed by chemiluminescence detection (*8*, *22*).

### Ribosomal RNA analysis

Plants at the booting stage were used for ribosome isolation, based on a protocol adapted from Mustroph *et al.* and modified by Li *et al.* (*23*, *24*). Tissue powder (2 g) was added to 10 ml polysome extraction buffer (PEB) and homogenized [200 mM tris-HCl (pH 9), 200 mM KCl, 25 mM EGTA, 35 mM MgCl_2_, 0.2% (v/v) Brj-35, 0.2% (v/v) Triton X-100, 0.2% (v/v) Igepal CA630, 0.2% (w/v) polyoxyethylene 10 tridecyl ether, 5 mM dithiothreitol (DTT), cycloheximide (100 mg/ml), chloramphenicol (100 mg/ml), and a complete proteinase inhibitor cocktail]. The homogenate was passed through two layers of Miracloth followed by centrifugation at 10,000*g* for 15 min at 4°C. The supernatant was transferred and filtered through two layers of Miracloth. The filtrate was layered on top of a 1.7 M sucrose cushion and centrifuged at 170,000*g* for 3 hours at 4°C. The ribosome pellet was resuspended in reaction buffer [20 mM tris-HCl (pH 8.0), 150 mM NaCl, 5 mM MgCl_2_, 1 mM DTT, cycloheximide (100 g/ml), and chloramphenicol (100 g/ml)].

Ribosome profiling was adapted from Ingolia *et al.* (*25*). In total, 600 μl of ribosome solution with an absorbance at 260 nm of 2 was used for digestion at room temperature for 1 hour with the addition of 15 μl of ribonuclease I (RNase I; Invitrogen, catalog no. AM2294). The reaction was stopped by adding 20 μl of RNase inhibitor (Invitrogen, catalog no. AM2694). Monosomes were collected by sucrose density gradient centrifugation at 50,000 rpm (Beckman, SW55.1 rotor) for 1.5 hours at 4°C. The synthesized complementary DNA (cDNA) was used as template for PCRs. The sequences of PCR primers are given in table S4.

### Translation efficiency

mRNA expression of *OsNRT2.3a* and *OsNRT2.3b* levels was determined for HTNE-1 and HTNE-2 by qRT-PCR. Ribosome RNA expression abundance of *OsNRT2.3a* and *OsNRT2.3b* was determined for HTNE-1 and HTNE-2 by PCR. ImageJ (National Institutes of Health) was used for gel scanning and quantification of ribosome RNA and protein. The ratio of ribosome RNA or protein abundance of *OsNRT2.3a* and *OsNRT2.3b* quantified by ImageJ to mRNA of *OsNRT2.3a* and *OsNRT2.3b* was taken as translation efficiency.

### Determination of nitrogen uptake using ^15^N

The lines were grown in international rice research institute (IRRI) nutrient solution for 3 weeks and then were deprived of nitrogen for 3 days at different temperatures in a temperature-controlled incubator. Four temperature treatments (night/day) were tested: 25°/32°C (control conditions), 32°/25°C, 25°/25°C, and 32°/32°C. Plants were rinsed in 0.1 mM CaSO_4_ for 1 min, then transferred separately to the IRRI nutrient solution containing 0.1 mM Ca(NO_3_)_2_ for 5 min, and then rinsed again with 0.1 mM CaSO_4_ for 1 min. All plants were heated to 105°C for 30 min to inactivate enzymes. The roots and shoots were separated and dried in an oven at 70°C for 7 days. The dry samples were ground to powder, and about 1 mg of the powder of each sample was analyzed using a DELTA V Advantage isotope mass spectrometer. Influx of ^15^NO_3_^−^ was calculated from the ^15^N concentrations of the roots.

### sRNA structure predictions

3D models for sRNAs were built at 25° and 32°C using the Build Biopolymer module of Schrödinger software Suite 2022-1. Protonation states at pH 7.0 were then assigned by PROPKA, and the hydrogen bond network was optimized. Last, the sRNA structure was optimized by minimizing molecular mechanical energy with OPLS_2005 force field parameters (*26*).

### MD simulations

MD simulations of the sRNA were performed with the Desmond program (*27*) using OPLS_2005 force field parameters. The sRNA structure was placed inside an orthorhombic box of 75.25 Å by 68.96 Å by 58.34 Å whose sides were at least 15 Å away from the nearest surface of sRNA with transferable interatomic potential with three points model (TIP3P) water molecules. The total charge of the entire system was neutralized by adding Na^+^ or Cl^−^ ions. An equilibration state was achieved by a short MD run before each main MD simulation using the Relax module in Desmond. The main MD simulations were performed in isobaric-isothermal ensembles with a pressure of 1 bar at 25° and 32°C. The total MD simulation time was 600 ns, and the trajectories were saved at intervals of 100 ps.

### sRNA and mRNA binding experiments

Binding analyses of sRNA with mRNA were carried out on 10% nondenaturing polyacrylamide gels (native gels). RNAs were dissolved in buffer containing 150 mM KCl, 40 mM tris-HCl (pH 7.2 at 37°C), and 8 mM MgCl_2_ at 37°C. Loading buffer [1 μl of 40% (v/v) glycerol and 1% (w/v) blue dextran] was mixed with 2 μl of RNA samples (0.1 μM mRNA and 0.5 μM sRNA in the buffer). Gels were stained with SYBR Gold (PerkinElmer Life Sciences) and 6-Carboxyfluorescein (FAM) independently and imaged using a fluorescence image scanner (fujifilm, FLA-5100). Before the measurements, the samples were heated to 80°C, cooled to experiment temperatures (4°, 25°, 37°, or 50°C) at a rate of −2°C min^−1^, and incubated at defined experimental temperatures for 30 min.

### Overexpression of sNRT2.3-1/2 in rice plants

The coding sequences of sNRT2.3-1 and sNRT2.3-2 were synthesized and cloned into the expression vector pTCK303. Briefly, the vector pTCK303 and the sNRT2.3-1 and sNRT2.3-2 synthetic fragments were digested with Kpn I and Hind III (Promega, Madison, WI, USA) at 37°C. DNA was purified using the QIAquick Gel Extraction Kit (QIAGEN, Valencia, CA, USA) and ligated to generate the expression vector pTCK303-sNRT2.3-1 and pTCK303-sNRT2.3-2. These vectors were transformed into rice *japonica* and Nip (cultivar Nip, HTNE-1 type) via Agrobacterium (*Agrobacterium tumefaciens*)–mediated infection.

### Generation of sNRT2.3-1 target mimic lines

The sRNA target mimic construct was generated according to the method described by Franco-Zorrilla *et al.* (*28*). Three artificial mimicry sequences (bATG, aATG as negative control, and mATG) with the CUA mismatch loop were inserted around the middle of the reverse complementary sequence of sRNA2.3-1. For bATG, the CUA (mismatch loop) was inserted before the CAU (the reverse complement of AUG); for aATG, the CUA was inserted after the CAU; and for mATG, the CUA was inserted within the CAU sequence complementary to sNRT2.3-1. These artificial mimicry sequences were then engineered into the *IPS1* gene, cloned into pGEM-T Easy vector (Promega), and used as a PCR template. The *IPS1* sequences harboring each artificial mimicry sequence (bATG, aATG, and mATG) were amplified using nested PCR. These fragments were then cloned into the pCUB vector (Sequence ID no. 24) and placed under the control of the rice *Ubiquitin* promoter via an In-Fusion reaction. The resulting vectors were transformed into rice *japonica* and Nip (HTNE-1 type) via Agrobacterium-mediated infection. Positive transgenic plants were used for the different N treatments for field experiments.

### Quantitative reverse transcription polymerase chain reaction

Total RNA was extracted separately from the roots and shoots of seedlings using TRIzol reagent (Thermo Fisher Scientific). Reverse transcription of 1.0-μg total RNA into first-strand cDNA was performed with a reverse transcription kit, and the synthesized cDNA was used as a template for qPCRs, with each biological replicate being analyzed in three technical replicates with an AceQ qPCR SYBR Green Master Mix kit (Vazyme Biotech, catalog no. Q311-02) on an Applied Biosystems (ABI) Plus Real-Time PCR System.

### Target-specific DMS-MaPseq

Target-specific DMS-Multiplexed Analysis of Projections by Sequencing (MaPseq) was performed as described (*29*–*31*). Following deoxyribonuclease I treatment, 3 μg of non-DMS–treated sample or 6 μg of DMS-treated sample was mixed with 0.5 μl of gene-specific RT primers mixture (table S1). The mixture was precipitated and resuspended in 10 μl of tris-KCl solution [50 mM KCl and 10 mM tris-HCl (pH 7.5)]. The solution was heated to 75°C for 3 min, followed by incubation at 57°C for 15 min. Four microliters of 5× first-strand buffer, 1 μl of 0.1 M DTT, 1 μl of SUPERase-In RNase inhibitor (Thermo Fisher Scientific), 1 μl of RNase-free H_2_O, and 1 μl of TGIRT-III (Ingex, catalog no. TGIRT50) were added to the solution. After incubation at room temperature for 30 min, 2 μl of 10 mM deoxynucleotide triphosphates was mixed into the solution, and reverse transcription was conducted at 60°C for 2.5 hours. Then, 2 μl of 2.5 M NaOH was added to stop the reaction and degrade RNA. The mixture was incubated at 95°C for 3 min and neutralized by adding HCl. Next, the cDNA was cleaned with RNAClean XP beads (Beckman). The targets were amplified with KODFX hot start DNA polymerase (Toyobo) using gene-specific primers (table S3). PCR products were gel-purified, and their concentration was adjusted according to band intensity. The library was constructed mainly following a published protocol (*29*) with New England Biolabs (NEB) adaptors and primers for Illumina (NEB). The libraries were quantified using Agilent TapeStation before sequencing as 2 × 250-bp paired-end reads on an Illumina NovaSeq 6000 instrument at Novagene.

### In vivo DMS modification

DMS treatment was performed as described (*29*, *31*, *32*). Two-week-old rice (*O. sativa* spp. Nip) seedlings grown in hydroponic nutrient solution were harvested and immersed in 20 ml of 1× DMS reaction buffer [40 mM Hepes (pH 7.5), 100 mM KCl, and 0.5 mM MgCl_2_]. Each sample then received 200 μl of DMS (MACKLIN, catalog no. D824267) to a final concentration of 1% (v/v). For the no DMS treatment control, an equivalent volume of diethyl pyrocarbonate (DEPC)–treated water was added to the reaction buffer. Treatment was performed for 15 min at 28°C with shaking at 250 rpm. To stop the DMS reaction, 6 ml of β-mercaptoethanol (Sigma-Aldrich, catalog no. M6250) was added to a final concentration of 23%. The mixture was incubated under vacuum for 5 min at room temperature. The samples were then washed three times with 50 ml of DEPC-treated water each, frozen in liquid nitrogen, and ground to fine powder.

### Sequencing analysis

Data analysis was performed as described previously (*29*). For filtering the raw fastq files, Trimmomatic (www.usadellab.org/cms/?page=trimmomatic) was used to remove adapter sequences (*33*). A sliding window trimming approach was used to filter the reads with quality scores lower than 20. Next, 2 nt from the 5′ end of each read was trimmed (*30*), and the reads over 200 bp were kept for further analysis. Reads were aligned to the reference sequence, *O. sativa* L. spp. *japonica*, using TopHat (*34*) (https://ccb.jhu.edu/software/tophat/index.shtml) with parameter setting: --library-type fr-unstranded --no-novel-juncs -N 25 --read-gap-length 7 --read-edit-dist 25 --max-insertion-length 5 --max-deletion-length 5 -g 3 --no-mixed. Uniquely mapped reads were extracted from the bam file. After discarding mismatches located within 3 nt of an InDel, the DMS signal was calculated for each adenine (A) and cytosine (C) nucleotide as mismatch/sequencing depth.

### Genomic DNA deep sequencing of *OsNRT2.3*

Genomic DNA was extracted with a DNA Quick Plant System (Tiangen Biotech, Beijing, China). Two pair of primers were used, *p*1505-F/R and *OsNRT2.3*-F/R, to amplify the promoter and coding region of *OsNRT2.3*, respectively. The sequences of natural accessions were aligned to the reference sequence of *O. sativa* L. spp. *japonica*, while the sequences obtained from tilling lines were aligned to the sequence of cultivar ZH11 using DNAMAN. The sequences of PCR primers are given in table S3.

### sRNA sequencing

Samples were collected for the first leaf blade, culm, and panicles at the anthesis stage for sRNA sequencing and combined. Total RNA was used to prepare the sequencing libraries, which were sequenced by KangChen Bio-tech, Shanghai, China. Raw sequencing reads generated from sRNA sequences were trimmed by removing adapter sequences. sRNAs of 24 nt in length were selected and aligned to the reference genome of *O. sativa* spp. *japonica* IRGSP-1.0.

### Methylated DNA immunoprecipitation sequencing

Methylated DNA immunoprecipitation (MeDIP) sequencing was performed by KangChen Bio-tech, Shanghai, China, using genomic DNA extracted from the same samples used for sRNA sequencing. Sequencing library preparation and data analysis were performed as described by Fan *et al.* (*35*). Clean sequencing reads were aligned to the reference genome of *O. sativa* spp. *japonica* IRGSP-1.0. MeDIP peaks were identified by MACS2 (*36*), and statistically significant MeDIP-enriched regions (peaks) were identified by comparison to a Poisson background model (with a cutoff *q* value = 10^−2^). Differentially methylated regions in the MeDIP dataset were identified by diffReps (*37*) (*P* < 0.0001). The MeDIP dataset generated in this work is available in the National Center for Biotechnology Information Gene Expression Omnibus (NCBI GEO) repository under accession number GSE94319.

### Northern blot analysis

Total RNA was isolated from 14-day-old seedlings using TRIzol reagent (TIANGEN, DP405-02) according to the manufacturer’s instructions. Three micrograms of total RNA was separated on a 1.2% (w/v) agarose/formaldehyde gel or a 15% (w/v) acrylamide/7 M urea gel and transferred to a Hybond N^+^ membrane (GE Healthcare) by capillary elution or electro transfer, respectively. Probes labeled with Digoxigenin-dUTP (DIG-dUTP) (0.5 pM) using the DIG Oligonucleotide Tailing Kit (Roche) were hybridized to the membrane according to the manufacturer’s protocol. The membrane was washed and exposed to a storage phosphor screen (GE Healthcare), and then signals were detected on a Typhoon TRIO scanner (GE Healthcare).

## Notes

View/request a protocol for this paper from *Bio-protocol*.
