# Supplementary material for: High-temperature adaptation of an OsNRT2.3 allele is thermoregulated by small RNAs
Source: Sci Adv. 2022 Nov 23;8(47):eadc9785. doi: 10.1126/sciadv.adc9785 (PMC9683703; doi:10.1126/sciadv.adc9785)
Supplement: Supplementary file 1 — Figs. S1 to S22 Tables S1 to S4 [file sciadv.adc9785_sm.pdf]

Science Advances
AAAS

Supplementary Materials for

High-temperature adaptation of an *OsNRT2.3* allele is thermoregulated by small RNAs

Yong Zhang *et al.*

Corresponding author: Xiaorong Fan, xiaorongfan@njau.edu.cn

Sci. Adv. **8**, eadc9785 (2022)
DOI: 10.1126/sciadv.adc9785

This PDF file includes:

Figs. S1 to S22
Tables S1 to S4

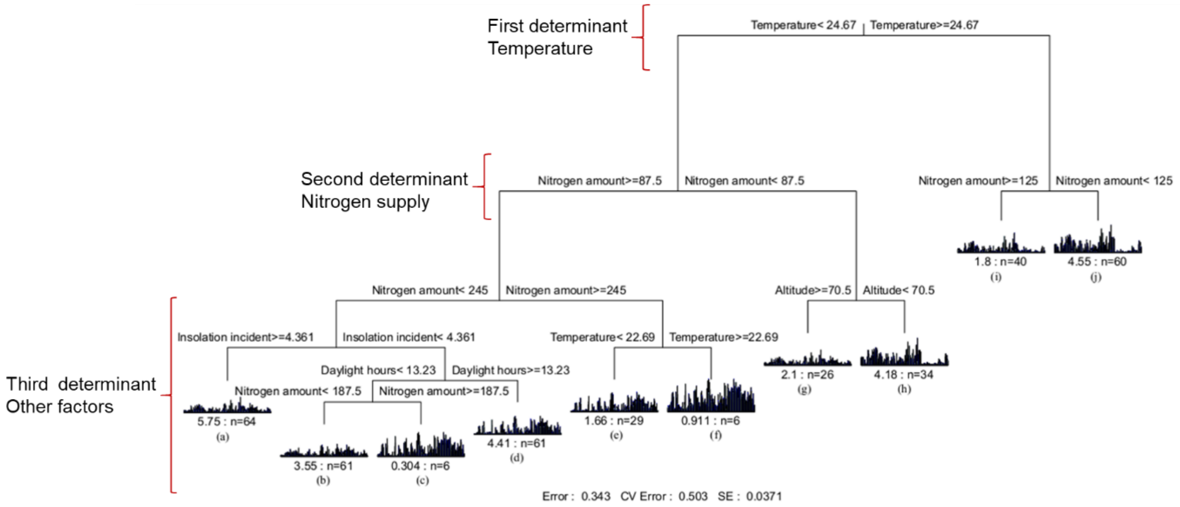

Figure S1. Multivariate regression tree (MRT) for the collected data related to CFEN in rice

MRT analysis was conducted to determine which factors were directly influencing the CFEN of different rice varieties. Each split in the graph is represented graphically as a branch in the tree. Bar plots show the multivariate means estimated for each branch. The numbers of samples included in that splits are shown under bar plots. The analysis indicated that nighttime temperature was the first (the most important) determinant of CFEN in different rice varieties. Whereas, the second most important determinant of CFEN in different rice varieties was the nitrogen supply condition. Then, it was followed by other factors such as insolation, daylight hours and altitude.

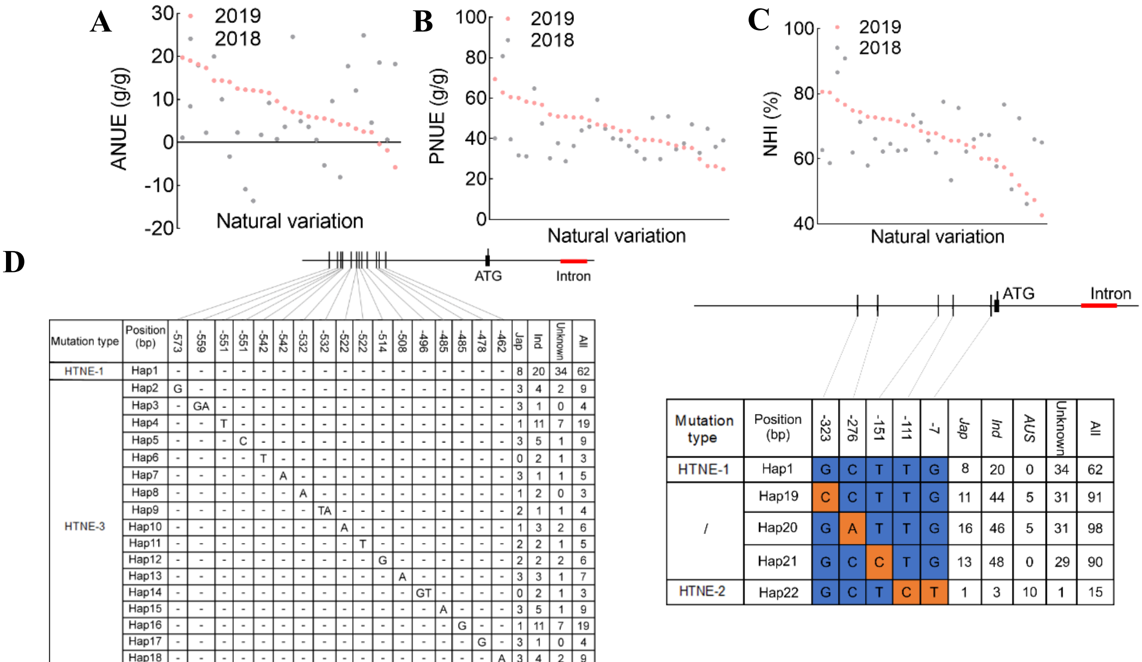

D

ATG
Intron

Mutation type	Position (bp)	-573	-559	-551	-551	-542	-542	-532	-532	-522	-522	-514	-508	-496	-485	-485	-478	-462	Jap	Ind	Unknown	All
HTNE-1	Hap1	-	-	-	-	-	-	-	-	-	-	-	-	-	-	-	-	-	8	20	34	62
HTNE-3	Hap2	G	-	-	-	-	-	-	-	-	-	-	-	-	-	-	-	-	3	4	2	9
	Hap3	-	GA	-	-	-	-	-	-	-	-	-	-	-	-	-	-	-	3	1	0	4
	Hap4	-	-	T	-	-	-	-	-	-	-	-	-	-	-	-	-	-	1	11	7	19
	Hap5	-	-	-	C	-	-	-	-	-	-	-	-	-	-	-	-	-	3	5	1	9
	Hap6	-	-	-	-	T	-	-	-	-	-	-	-	-	-	-	-	-	0	2	1	3
	Hap7	-	-	-	-	-	A	-	-	-	-	-	-	-	-	-	-	-	3	1	1	5
	Hap8	-	-	-	-	-	-	A	-	-	-	-	-	-	-	-	-	-	1	2	0	3
	Hap9	-	-	-	-	-	-	-	TA	-	-	-	-	-	-	-	-	-	2	1	1	4
	Hap10	-	-	-	-	-	-	-	-	A	-	-	-	-	-	-	-	-	1	3	2	6
	Hap11	-	-	-	-	-	-	-	-	-	T	-	-	-	-	-	-	-	2	2	1	5
	Hap12	-	-	-	-	-	-	-	-	-	-	G	-	-	-	-	-	-	2	2	2	6
	Hap13	-	-	-	-	-	-	-	-	-	-	-	A	-	-	-	-	-	3	3	1	7
	Hap14	-	-	-	-	-	-	-	-	-	-	-	-	GT	-	-	-	-	0	2	1	3
	Hap15	-	-	-	-	-	-	-	-	-	-	-	-	-	A	-	-	-	3	5	1	9
	Hap16	-	-	-	-	-	-	-	-	-	-	-	-	-	-	G	-	-	1	11	7	19
	Hap17	-	-	-	-	-	-	-	-	-	-	-	-	-	-	-	G	-	3	1	0	4
	Hap18	-	-	-	-	-	-	-	-	-	-	-	-	-	-	-	-	A	3	4	2	9

ATG
Intron

Mutation type	Position (bp)	-323	-276	-151	-111	-7	*Jap*	*Ind*	*AUS*	Unknown	All
HTNE-1	Hap1	G	C	T	T	G	8	20	0	34	62
/	Hap19	C	C	T	T	G	11	44	5	31	91
	Hap20	G	A	T	T	G	16	46	5	31	98
	Hap21	G	C	C	T	G	13	48	0	29	90
HTNE-2	Hap22	G	C	T	C	T	1	3	10	1	15

Figure S2. Nitrogen use efficiency of natural varieties

A, Agronomic nitrogen use efficiency (ANUE) of 2018 and 2019. The orange circle is the ANUE of 2019, and grey circle is 2018. **B**, Physiological nitrogen use efficiency (PNUE, the ratio of yield/N amount in shoot) of 2018 and 2019. **C**, Nitrogen harvest index (NHI, the ratio of N amount in grain/N amount in shoot) of 2018 and 2019. Each natural variety has five replications. The maximum and minimum temperature of 2018 is higher than 2019 (Detailed temperature show in Fig. 1A). In 2019 ANUE and PNUE was generally higher than in 2018. **D**, Alleles analysis of *OsNRT2.3* among 239 natural rice varieties. Analysis of the sequences of a 750 region upstream of the translation initiation codon of *OsNRT2.3* (noted from here as region from -1 to -750 bp), allowed us to classify the 239 rice accessions into 22 different haplotypes. These alleles were further grouped into 3 main haplotypes, HTNE-1 containing no SNPs respect to the Nipponbare reference genome, HTNE-2 with two conserved SNPs at -111 bp and -7 bp upstream of the *OsNRT2.3* ATG and HTNE-3 containing an insertion and SNPs in the promoter of *OsNRT2.3*. HTNE, High Temperature resistant and Nitrogen Efficient.

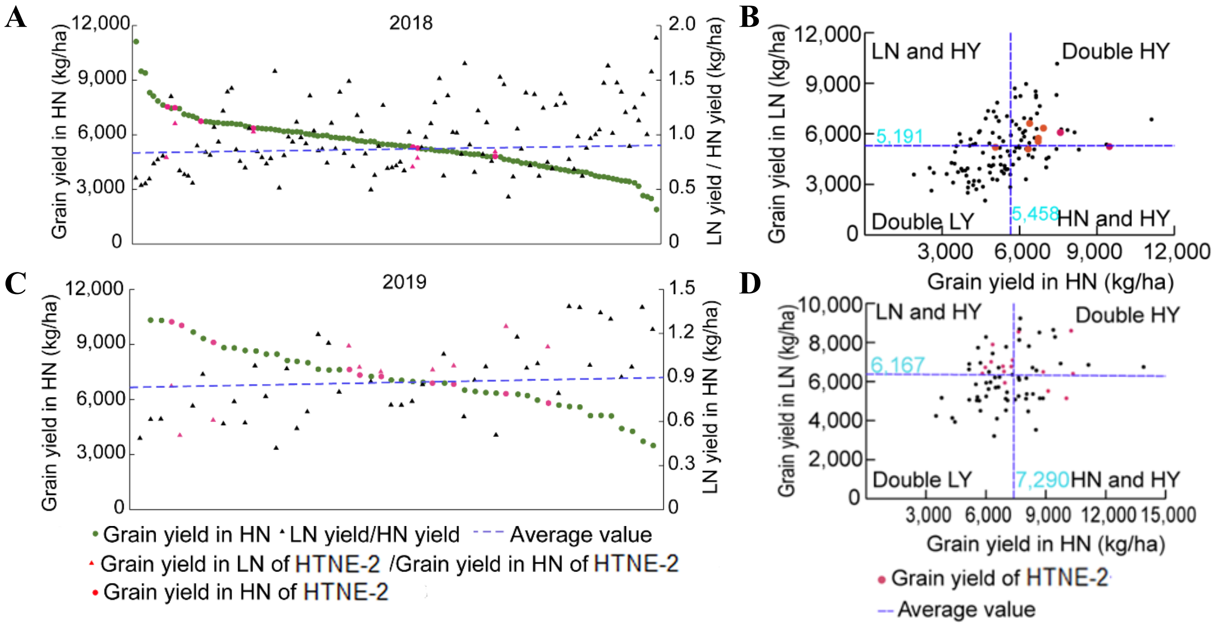

Figure S3. The grain yield of natural varieties under high nitrogen and low nitrogen condition

High-nitrogen yield and the ratio of low-nitrogen yield to high-nitrogen yield of natural varieties in 2018 (**A**) and 2019 (**C**). The high nitrogen is 120 kg-N/ha, low nitrogen is 60 kg-N/ha. Green circle, grain yield in high nitrogen (HN); black triangle, low-nitrogen (LN) yield to high-nitrogen yield; red dotted lines represent the average value; blue circle, grain yield in high nitrogen of HTNE-2; blue triangle, the ratio of grain yield in LN and HN of HTNE-2. Each natural variety have five replications. The yield of natural varieties under High Nitrogen (HN) and Low Nitrogen (LN) in 2018 (**B**) and 2019 (**C**). LY and HY, high yield at low nitrogen; Double HY, high yield at low/high nitrogen; Double LY, low yield at low/high nitrogen; HN and HY, high yield at high nitrogen. The circle represents natural varieties, and the red circle represents HTNE-2. The blue dotted lines represent the average value. Interestingly, 81.5% (2018, high temperature) and 27.9% (2019, low temperature) of the HTNE-2 accessions were high yielding in both low-nitrogen and high-nitrogen conditions. During the high temperature year (2018), the HTNE-2 shows more supercity than that in low temperature year.

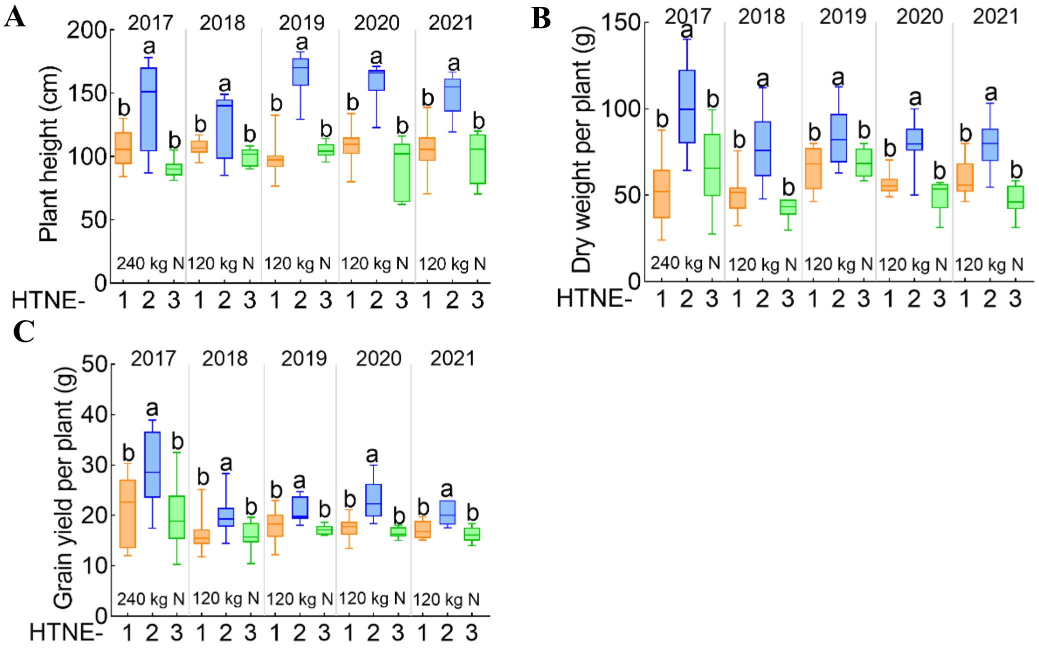

Figure S4. The phenotype of HTNE-1, HTNE-2 and HTNE-3

Plant height (**A**), dry weight per plant (**B**) and grain yield per plant (**C**) of HTNE-1, HTNE-2 and HTNE-3 in five years. HTNE-1, *OsNRT2.3* alleles without SNPs in natural varieties; HTNE-2, *OsNRT2.3* alleles which co-exist the SNPs at position -111 upstream of the *OsNRT2.3* ATG (T to C), and at position -7 (G to T); HTNE-3, *OsNRT2.3* alleles which have insertion base and SNPs. Significant differences between each other are indicated by different letters ($p< 0.05$; unpaired two-tailed Student's *t*-test).

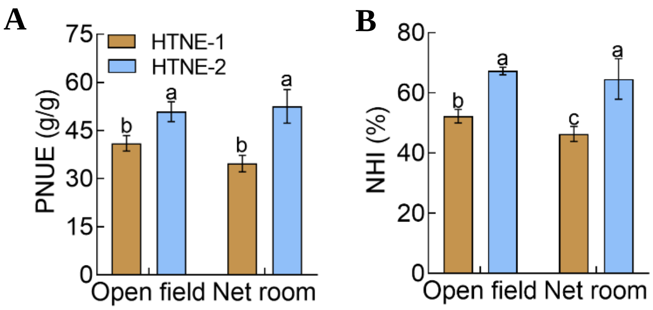

Figure S5. The PNUE and NHI of HTNE-1 and HTNE-2 in the open field and net room
The lines of HTNE-1 and HTNE-2 were grown in the open field and net room in Nanjing, China. (**A**) Physiological nitrogen use efficiency (PNUE) of HTNE-1 and HTNE-2 in the open field and net room. (**B**) Nitrogen harvest index (NHI) of HTNE-1 and HTNE-2 in the open field and net room. HTNE-1, n=10 biological replicates. HTNE-2, n=6 biological replicates. Significant differences between each other are indicated by different letters ($p < 0.05$; unpaired two-tailed Student's *t*-test).

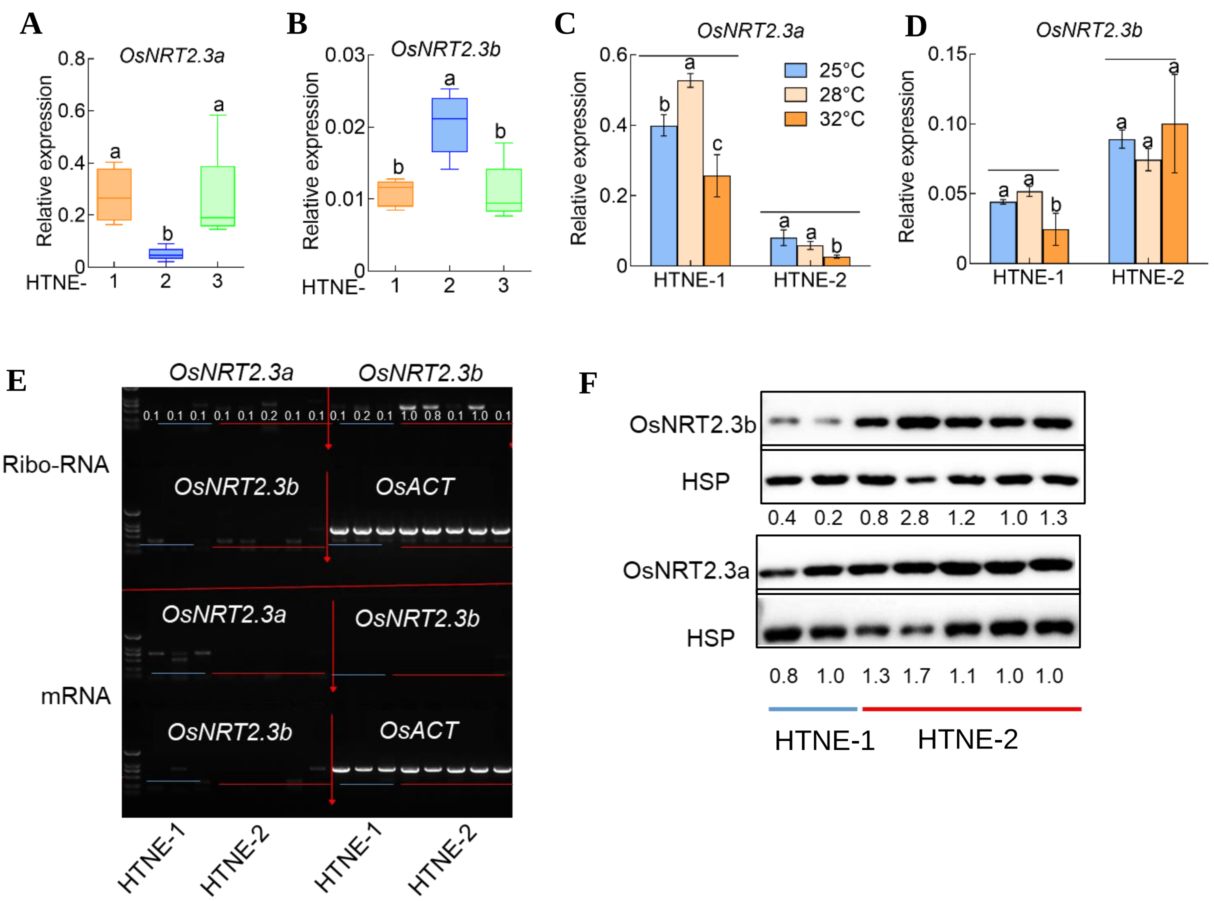

Figure S6. The translation of *OsNRT2.3a* and *OsNRT2.3b* in HTNE-1 and HTNE-2

The expression of *OsNRT2.3a* (**A**) and *OsNRT2.3b* (**B**) was analyzed in HTNE-1, HTNE-2 and HTNE-3 in the field condition at booting stage when the root temperature in soil was about 25 ℃ and the shoot temperature was about 28 ℃ sampling at 10:00 of day during the middle of July. Messager RNA expression of *OsNRT2.3a* (**C**) and *OsNRT2.3b* (**D**) in HTNE-1 and HTNE-2 under 25 ℃, 28 ℃ and 32 ℃ condition. **E,** The expression of *OsNRT2.3a* and *OsNRT2.3b* in ribosome RNA samples of HTNE-1 and HTNE-2 under 28 ℃. **F,** Western blot analysis of OsNRT2.3a and OsNRT2.3b protein of HTNE-1 and HTNE-2 with the same samples as used in (A), (B). The number which under the figure is relative expression. Data are shown as means ± SE (n=3 biological replicates). Significant differences between each other are indicated by different letters (p< 0.05; unpaired two-tailed Student's t-test).

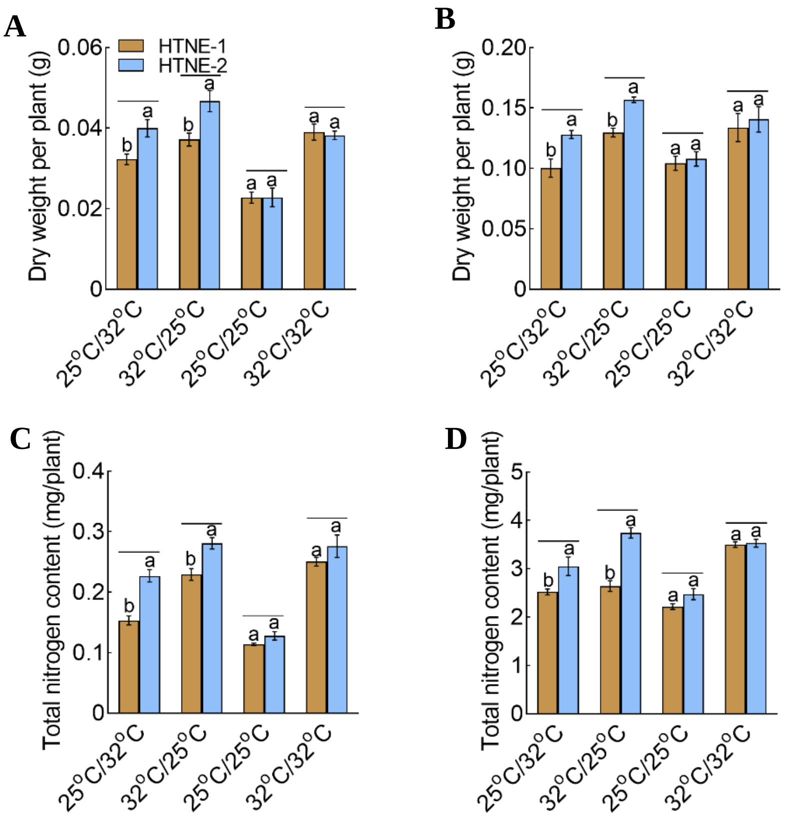

Figure S7. The phenotype of HTNE-1 and HTNE-2 in different temperature

The lines of HTNE-1 and HTNE-2 were planted in the temperature controlled incubator under 0.1 mM $Ca(NO_3)_2$ and different temperature treatment for one weak. Four temperature treatments (night/day) were set as 25 ℃/32 ℃ (control treatment), 32 ℃/25 ℃, 25 ℃/25 ℃ and 32 ℃/32 ℃. The dry weight of root (**A**) and shoot (**B**) were taken. The total nitrogen content of root (**C**) and shoot (**D**) of HTNE-1 and HTNE-2. Error bars: SE (n=3 biological replicates). Significant differences between each other are indicated by different letters. ($p < 0.05$; unpaired two-tailed Student's *t*-test).

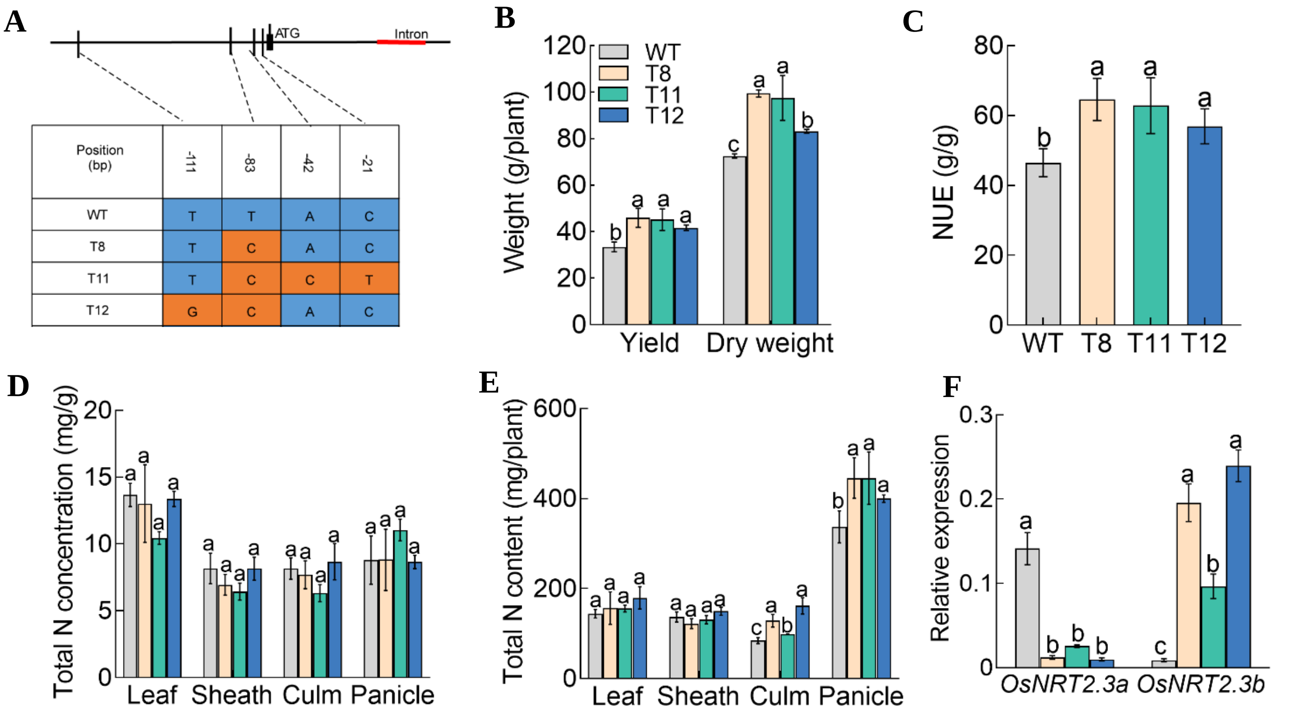

Figure S8. Nitrogen content in *OsNRT2.3* tilling lines at the maturity stage in the field
A, Nucleotide sequence of *OsNRT2.3* tilling lines. Wild type, Zhonghua 11 (WT, HTNE-1). T8, T11 and T12 having one to three-point mutations at positions -21, -42, -83 and -111 bp upstream of the *OsNRT2.3* ATG in Zhonghua 11. **B**, Grain yield and dry weight per plant for *OsNRT2.3* tilling lines and WT in the field. Dry weight mean values represent aboveground biomass, excluding grain yield. **C**, The NUE of WT, T8, T11 and T12. **D**, Total N concentration of different parts in the *OsNRT2.3* tilling lines. **E**, Total N content of different parts in the *OsNRT2.3* tilling lines. **F**, The expression of *OsNRT2.3a* and *OsNRT2.3b* in the tilling lines (T8, T11 and T12). Error bars: SE (n=5 biological replicates). Significant differences between the *OsNRT2.3* tilling lines and WT are indicated by different letters ($p < 0.05$; unpaired two-tailed Student's *t*-test).

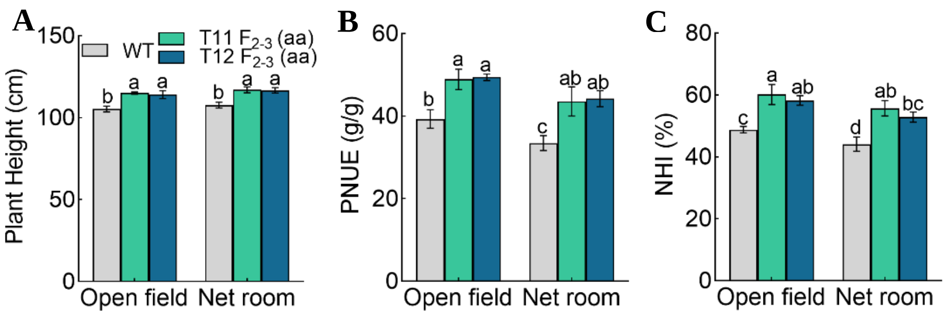

Figure S9. The phenotype of the *OsNRT2.3* tilling lines in the open field and net room

The *OsNRT2.3* tilling lines were grown in open field and net-room in Nanjing, China. **A**, The plant height of the *OsNRT2.3* tilling lines and wild type (WT) in the open field and net room. **B**, Physiological nitrogen use efficiency (PNUE) of the *OsNRT2.3* tilling lines and WT in the open field and net room. **C**, Nitrogen harvest index (NHI) of the *OsNRT2.3* tilling lines and WT in the open field and net room. Error bars: SE (n=5 biological replicates). Significant differences between each other are indicated by different letters ($p< 0.05$; unpaired two-tailed Student's *t*-test).

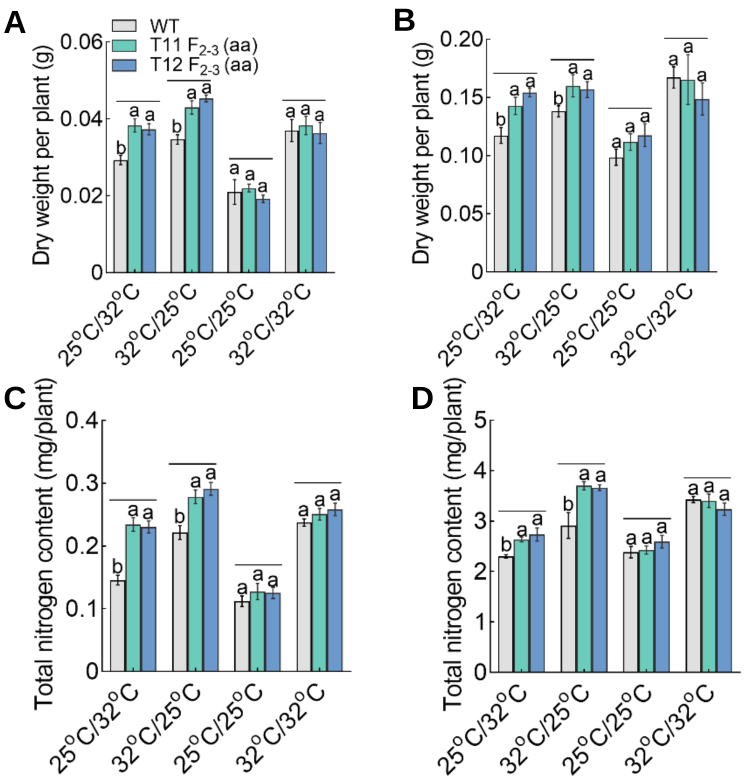

Figure S10. The phenotype of *OsNRT2.3* tilling lines in different temperature

The *OsNRT2.3* tilling lines and WT were planted in the temperature controlled incubator under 0.1 mM $Ca(NO_3)_2$ and different temperature treatment for one weak. Four temperature treatments (night/day) were set as 25°C/32°C (control treatment), 32°C/25°C, 25°C/25°C and 32°C/32°C. The dry weight of root (**A**) and shoot (**B**). Total nitrogen content of root (**C**) and shoot (**D**) of *OsNRT2.3* tilling lines and WT. Error bars: SE (n=3 biological replicates). Significant differences between each other are indicated by different letters. (p< 0.05; unpaired two-tailed Student's *t*-test).

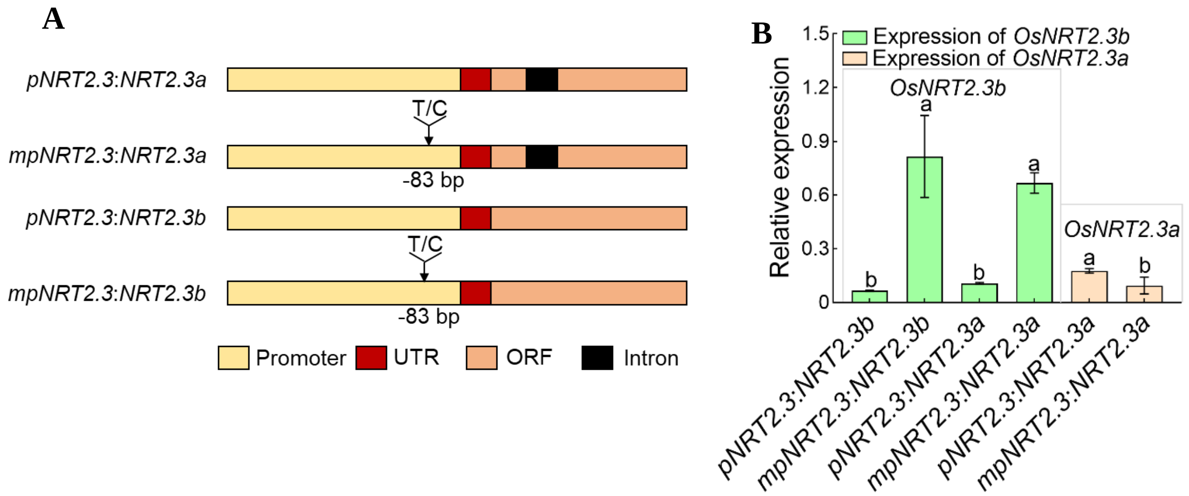

Figure S11. The -83 bp mutation upstream of *OsNRT2.3* ATG regulates the transcription of *OsNRT2.3a* and *OsNRT2.3b* in rice

A, Vectors construction diagram. Yellow frames are 1500 bp promoter of *OsNRT2.3a* or *OsNRT2.3b*. Red frames are 5'UTR. Brown frames are ORF of *OsNRT2.3a* and *OsNRT2.3b*. mp contains T to C mutation at position 83 bp upstream of the *OsNRT2.3* ATG. **B,** The expression of *OsNRT2.3a* and *OsNRT2.3b* in transgenic lines. *pNRT2.3*:*NRT2.3b*, the sequence without mutation at upstream of *OsNRT2.3* ATG promote *OsNRT2.3b*. *mpNRT2.3*:*NRT2.3b*, the sequence with mutation at 83 bp upstream of *OsNRT2.3* ATG promote *OsNRT2.3b*. *pNRT2.3*:*NRT2.3a*, the sequence without mutation at upstream of *OsNRT2.3* ATG promote *OsNRT2.3a*. *mpNRT2.3*:*NRT2.3a*, the sequence with mutation at 83 bp upstream of *OsNRT2.3* ATG promote *OsNRT2.3a*. Each data subtracts the background value in the wild type, Nip (*Nipponbare*, HTNE-1). Error bars: SE (n=3 biological replicates). Significant differences between transgenic lines are indicated by different letters. (p< 0.05; unpaired two-tailed Student's *t*-test).

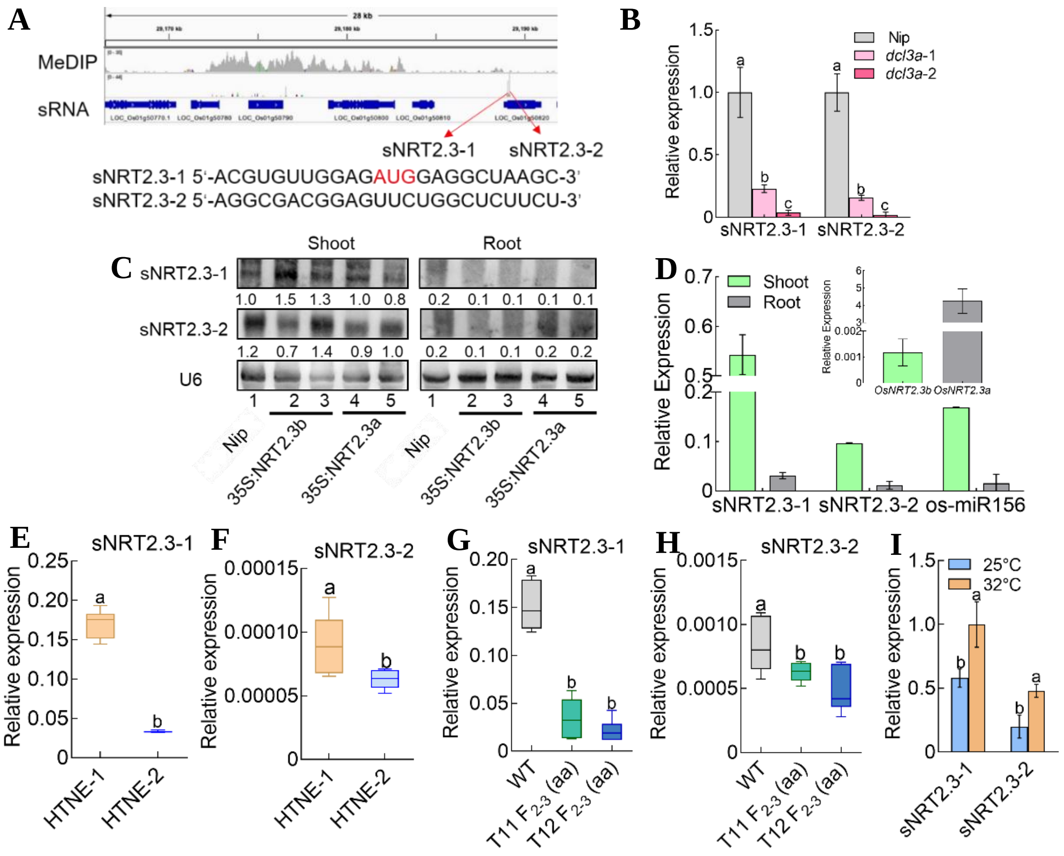

Figure S12. The expression pattern of sNRT2.3-1 and sNRT2.3-2

A, Position of sNRT2.3-1 and sNRT2.3-2 in the *OsNRT2.3* genomes. Sequenced sRNA libraries of rice plants grown under different conditions to create a new sRNA database (BioProject ID: PRJNA685618) of *Nipponbare* rice. We also determined the genomic DNA methylation profile of these region using MeDIP and the results showed there was no significant methylation activities at the position of sNRT2.3-1 and sNRT2.3-2. **B,** The expression of sNRT2.3-1 and sNRT2.3-2 in the *OsDCL3a* mutant lines and wild type. *OsDCL3a* responsible for the production of 24 nt sRNAs and the expression of sNRT2.3-1 and sNRT2.3-2 was significantly reduced in *OsDCL3a* mutant lines. **C,** Northern blot analysis the expression pattern of sNRT2.3-1 and sNRT2.3-2 in the shoot and root of *OsNRT2.3a* and *OsNRT2.3b* overexpressing lines and wild type (Nip, *Nipponare*, HTNE-1). Lane1 and 2, overexpression *OsNRT2.3a* lines (genetic background *Nipponare*). Lane3 and 4, overexpression *OsNRT2.3b* lines (genetic background *Nipponare*). Lane5, WT-N, Nipponare. **D,** The expression of *OsNRT2.3*, sNRT2.3-1, sNRT2.3-2 and osa-miR156 in shoot and root of rice. The HTNE-1 and HTNE-2 rice grown in the field, total RNA was extracted from HTNE-1 and HTNE-2 at booting stage sampled at the same time as the materials in Extended Data Fig. 4 and real-time PCR was carried out to determine the expression level of sNRT2.3-1 (**E**) and sNRT2.3-2 (**F**). The lines of T11/WT(aa), T12/WT(aa) and WT (Zhonghua11, HTNE-1) grown in the field, total RNA was extracted at booting stage and real-time PCR was carried out to determine the expression level of sNRT2.3-1 (**G**) and sNRT2.3-2 (**H**). **I**, The expression of sNRT2.3-1 and sNRT2.3-2 under different temperature. Error bars: SE (n=5 biological replicates). Significant differences between each other are indicated by different letters (p< 0.05; unpaired two-tailed Student's t-test).

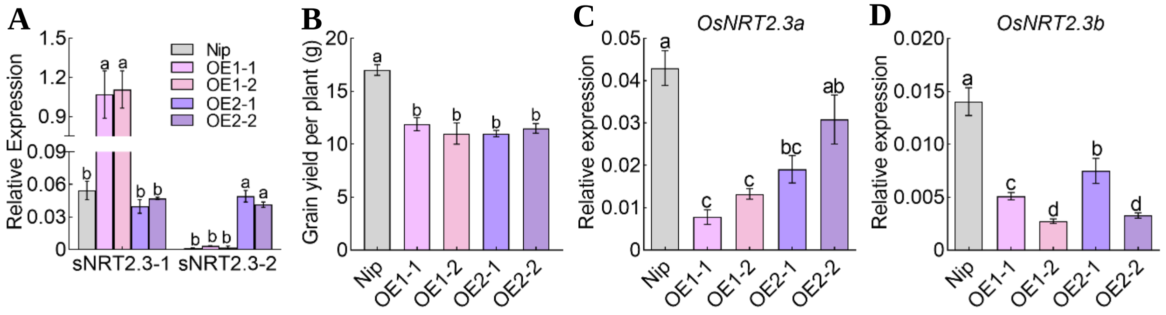

Figure S13. Characterization of sNRT2.3-1 and sNRT2.3-2 overexpressing lines and the expression of *OsNRT2.3a/b*

A, The expression of sNRT2.3-1/2 in sNRT2.3-1 overexpressing lines (OE1-1 and OE1-2) and sNRT2.3-2 overexpressing lines (OE2-1 and OE2-2). Error bars: SE (n=3 biological replicates). **B,** The yield was taken in the mature stage. Error bars: SE (n=5 biological replicates). The expression of *OsNRT2.3a* (**C**) and *OsNRT2.3b* (**D**) in sNRT2.3-1/2 overexpressing lines and wild type (*Nipponbare*, Nip). Significant differences between the transgenic lines and Nip are indicated by different letters ($p < 0.05$; unpaired two-tailed Student's t-test).

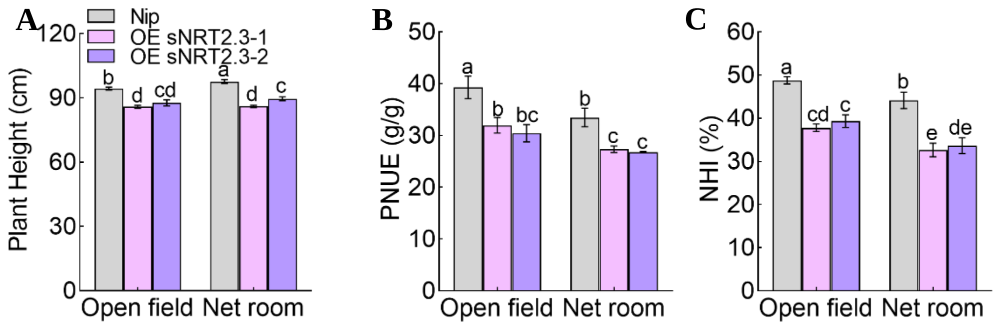

Figure S14. The phenotype of the sNRT2.3-1 and sNRT2.3-2 overexpressing lines in the open field and net-room

The sNRT2.3-1 and sNRT2.3-2 overexpressing lines were grown in the open field and net room in Nanjing, China. **A,** The plant height of the sNRT2.3-1 overexpressing lines (OE sNRT2.3-1), sNRT2.3-2 overexpressing lines (OE sNRT2.3-2) and wild type (Nip) in the open field and net room. **B,** Physiological nitrogen use efficiency (PNUE) of the OE sNRT2.3-1, OE sNRT2.3-2 lines and Nip in the open field and net room. **C,** Nitrogen harvest index (NHI) of the OE sNRT2.3-1, OE sNRT2.3-2 lines and Nip in the open field and net room. Error bars: SE (n=5 biological replicates). Significant differences between each other are indicated by different letters (p< 0.05; unpaired two-tailed Student's t-test).

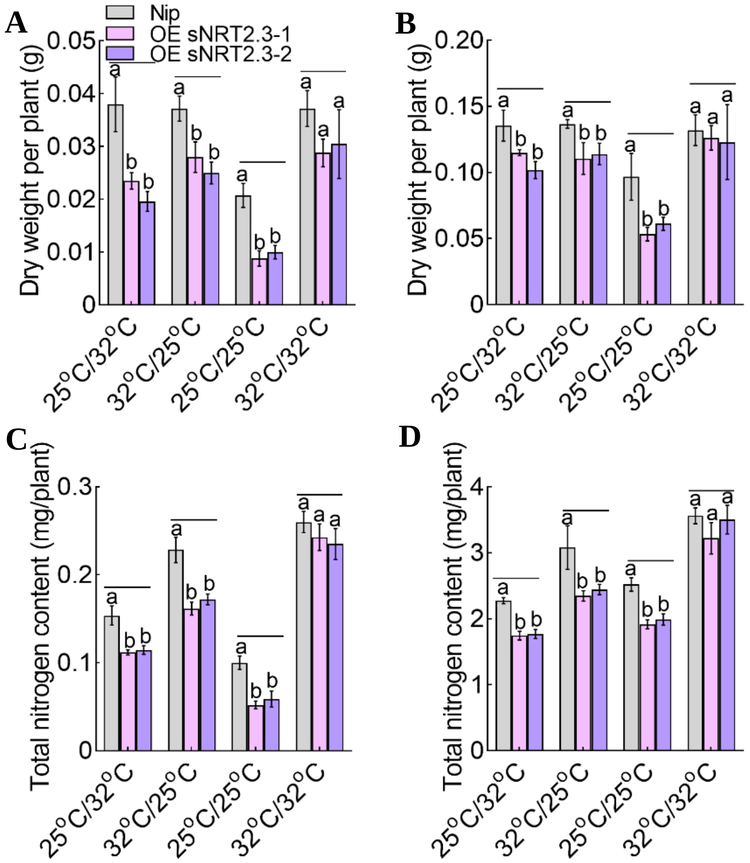

Figure S15. The phenotype of the sNRT2.3-1 and sNRT2.3-2 overexpressing lines in different temperature

The sNRT2.3-1 and sNRT2.3-2 overexpressing lines were planted in the temperature controlled incubator under 0.1 mM $Ca(NO_3)_2$ and different temperature treatment for one weak. Four temperature treatments (night/day) were set as 25°C/32°C (control treatment), 32°C/25°C, 25°C/25°C and 32°C/32°C. The dry weight of root (**A**) and shoot (**B**). Total nitrogen content of root (**C**) and shoot (**D**) of sNRT2.3-1 and sNRT2.3-2 overexpressing lines. Error bars: SE (n=3 biological replicates). Significant differences between each other are indicated by different letters. (p< 0.05; unpaired two-tailed Student's t-test).

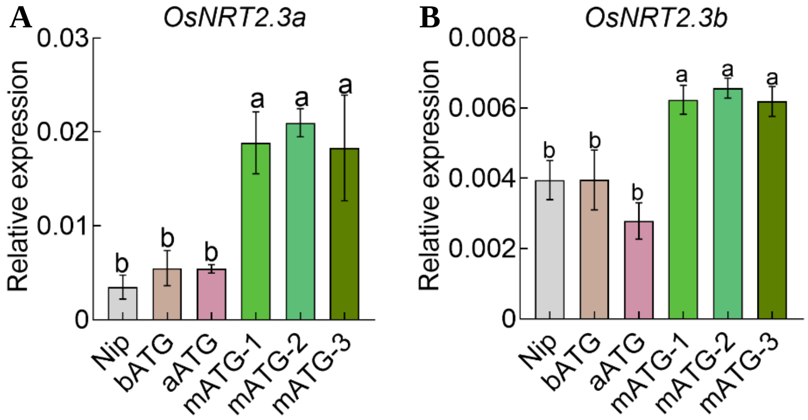

Figure S16. The expression of *OsNRT2.3a* and *OsNRT2.3b* in the sNRT2.3-1 mimic lines
The expression of *OsNRT2.3a* (**A**) and *OsNRT2.3b* (**B**) in the sNRT2.3-1 mimic lines. Error bars: SE (n=3 biological replicates). Significant differences between the lines and Nip are indicated by different letters. ($p < 0.05$; unpaired two-tailed Student's *t*-test).

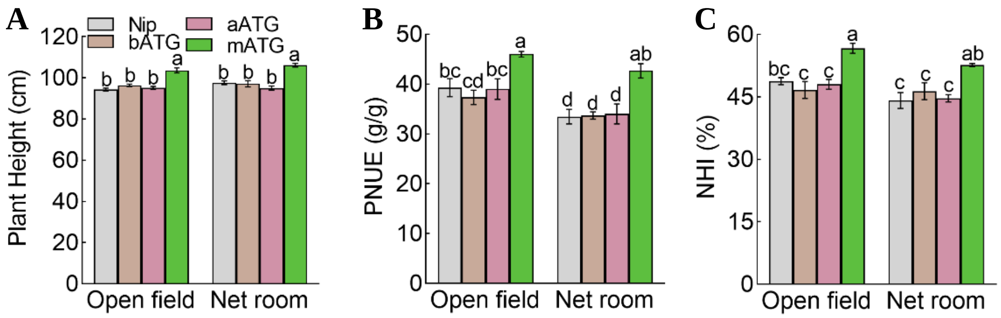

Figure S17. The phenotype of the target mimicry mutation lines in the open field and net-room

The target mimicry mutation lines were grown in the open field and net-room in Nanjing, China. **A,** The plant height of the target mimicry mutation lines (aATG, bATG and mATG) and wild type (Nip) in the open field and net-room. **B,** Physiological nitrogen use efficiency (PNUE) of the target mimicry mutation lines and Nip in the open field and net-room. **C,** Nitrogen harvest index (NHI) of the target mimicry mutation lines and wild Nip in the open field and net-room. Error bars: SE (n=5 biological replicates). Significant differences between each other are indicated by different letters ($p < 0.05$; unpaired two-tailed Student's *t*-test).

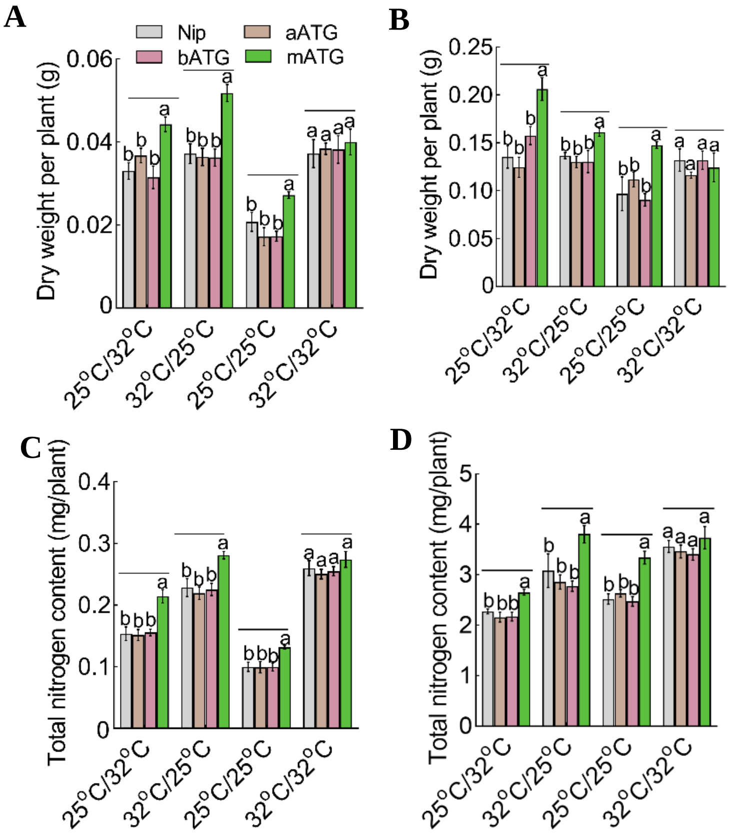

Figure S18. The phenotype of the target mimicry mutation lines in different temperature

The target mimicry mutation lines were planted in the temperature controlled incubator under 0.1 mM $Ca(NO_3)_2$ and different temperature treatment for one weak. Four temperature treatments (night/day) were set as 25 ℃/32 ℃ (control treatment), 32 ℃/25 ℃, 25 ℃/25 ℃ and 32 ℃/32 ℃. The dry weight of root (**A**) and shoot (**B**). Total nitrogen content of root (**C**) and shoot (**D**) of the target mimicry mutation lines. Error bars: SE (n=3 biological replicates). Significant differences between each other are indicated by different letters. ($p < 0.05$; unpaired two-tailed Student's *t*-test).

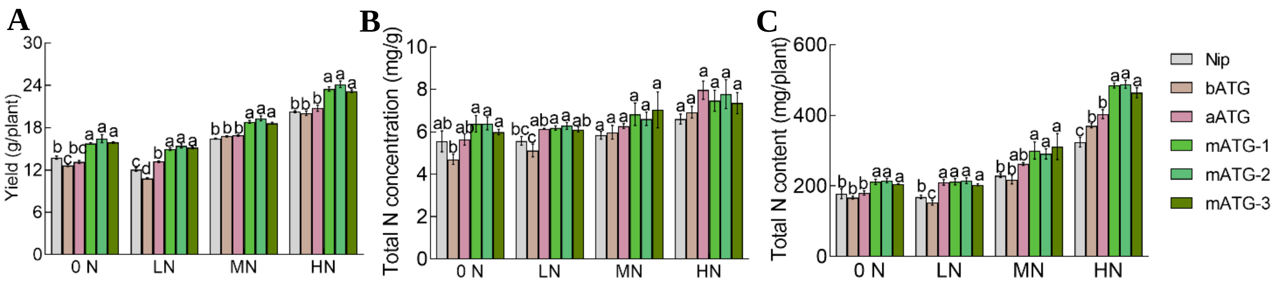

Figure S19. The nitrogen content of sNRT2.3-1 mimic lines in the field

The sNRT2.3-1 mimic lines were plant in the field with 0 nitrogen (0 N), low nitrogen (60 kg-N/ha, LN), middle nitrogen (120 kg-N/ha, MN) and high nitrogen (350 kg-N/ha, HN) treatment. At the mature stage, the yield (**A**), total nitrogen concentration (**B**) and total nitrogen content (**C**) was taken and analyses. Error bars: SE (n=5 biological replicates). Significant differences between the lines and Nip are indicated by different letters. ($p< 0.05$; unpaired two-tailed Student's *t*-test).

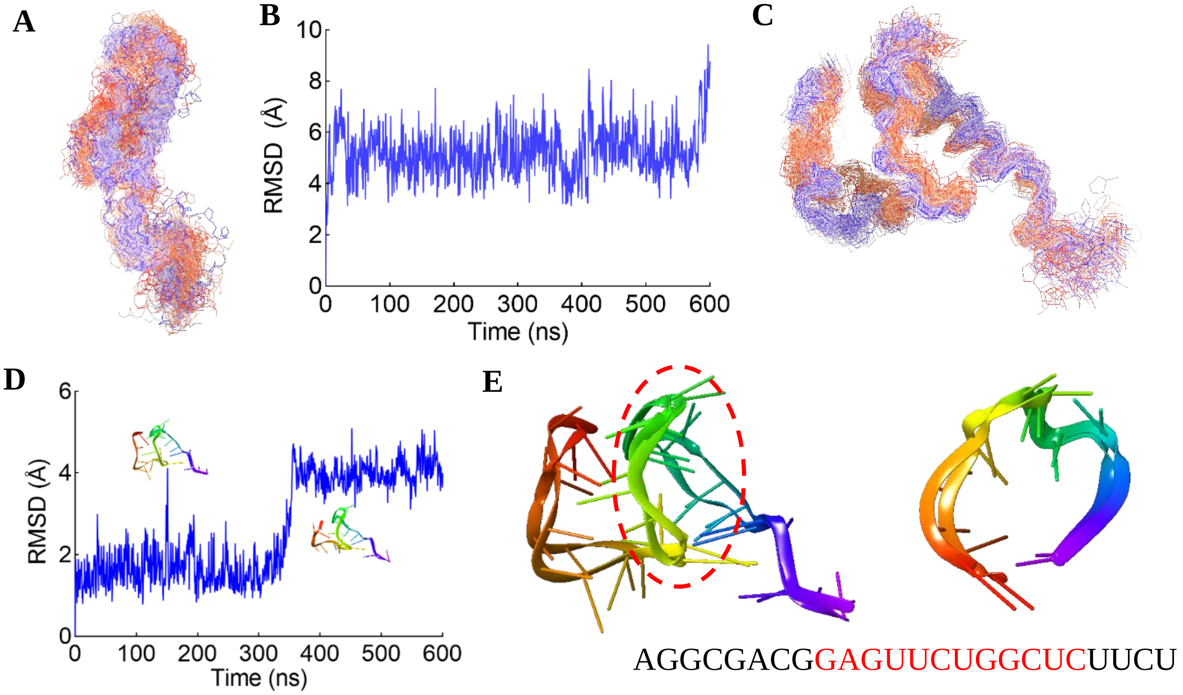

Figure S20. Extracted and sampled 101 sNRT2.3-2 structures from a 600 ns MD simulation at 25°C and 32°C

A, Extracted and sampled 101 sNRT2.3-2 structures from a 600 ns MD simulation at 25°C, aligned the 101sRNA backbone. **B,** The root mean square deviation (RMSD) values of sNRT2.3-2 25°C over a 600 ns MD simulation. **C,** Extracted and sampled 101 sNRT2.3-2 structures from a 600 ns MD simulation at 32°C, aligned the 101sRNA backbone. The RMSD values of sNRT2.3-2 32°C over a 600 ns MD simulation (**D**) and conformational changes at different times (0-300 ns, 400-500 ns). (**E**) Align the average structure of sNRT2.3-2 at 0-300 ns, 400-500 ns.

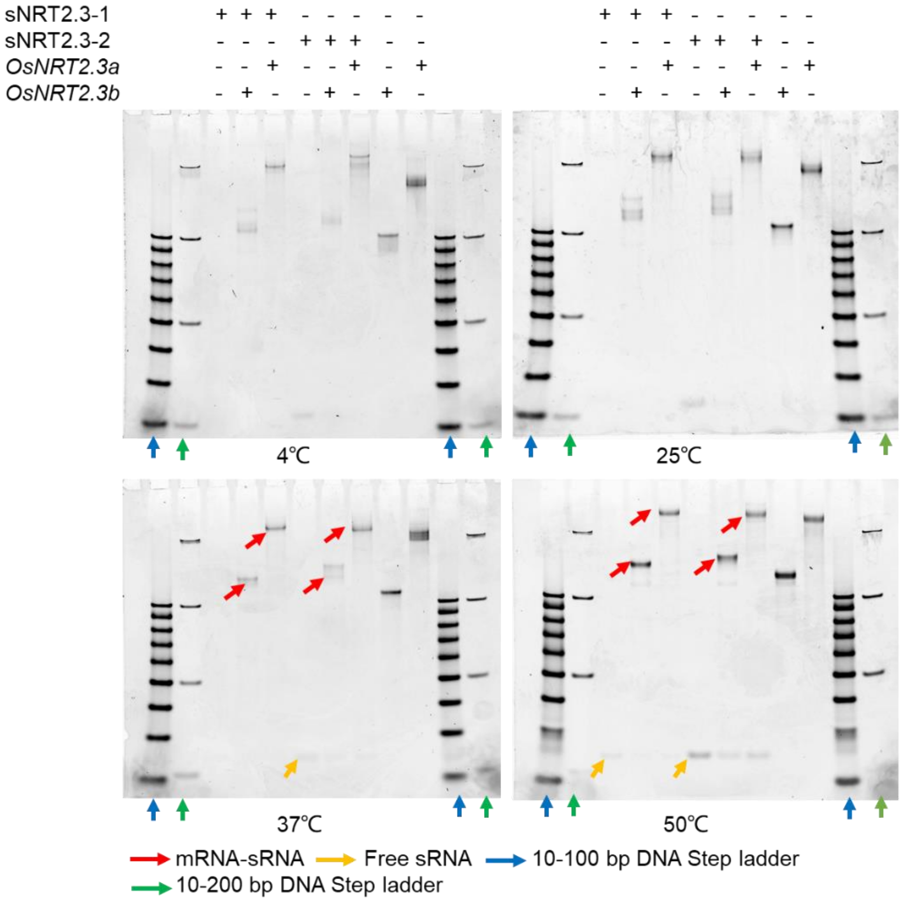

Figure S21. The binding capability of sNRT2.3-1 and sNRT2.3-2 to *OsNRT2.3a* and *OsNRT2.3b* at different temperature

The binding capability of sNRT2.3-1 and sNRT2.3-2 to *OsNRT2.3a* and *OsNRT2.3b* at 4℃, 25℃, 37℃ and 50℃. Gels were stained with SYBR® Gold (PerkinElmer Life Sciences). Red arrow, mRNA-sRNA complex; orange arrow, free sRNA; Blue arrow, 10-100 bp DNA Step Ladder; Green arrow, 10-200 bp DNA Step Ladder.

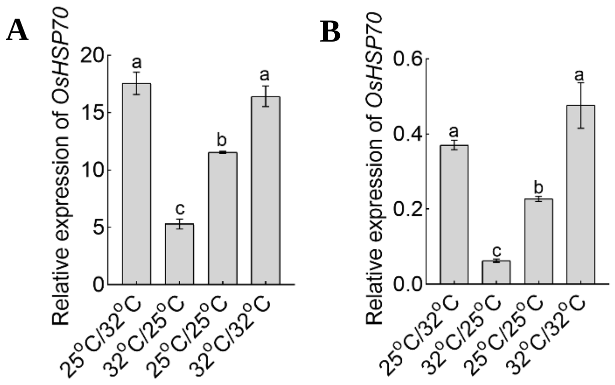

Figure S22. The expression of *OsHSP70* in Nip under different temperature

The rice, Nip, was grown in the temperature controlled incubator with different day and night temperature for one weak. Four temperature treatments (night/day) were set as 25 ℃/32 ℃ (control treatment), 32 ℃/25 ℃, 25 ℃/25 ℃ and 32 ℃/32 ℃. The expression of *OsHSP70*, the gene of heat shock protein 70, was taken. *OsActin* (**A**) and *OsTubulin* (**B**) as the endogenous control genes. Data are shown as means ± SE (n=3 biological replicates). Different lowercase letters indicate significant differences between each other (p < 0.05; unpaired two-tailed Student's t-test).

Table S1. The phenotype of BC_1F_{2-3} in the net room and open field

	Sequence from BC_1F_2			Data from BC_1F_{2-3}					
	ATG	ATG	Lines	Plant Height (cm)		PNUE		NHI (%)	
	-111 bp	-7 bp	NO.	Net room	Open field	Net room	Open field	Net room	Open field
HTNE-1 (♀)	T	G	5	95.34±2.03c(a)	91.30±1.85c(a)	35.24±2.48c(b)	48.49±0.84c(a)	40.67±1.13b(b)	54.31±1.80b(a)
HTNE-2 (♂)	C	T	5	155.00±3.50a(a)	152.00±6.5a(a)	50.02±2.38b(a)	55.25±4.87a(a)	58.71±2.26a(a)	65.00±1.42a(a)
HTNE-1^{TG}	T	G	61	146.25±3.06b(a)	141.60±2.52b(a)	33.74±2.19c(b)	42.02±1.67c(a)	41.16±0.85b(b)	52.98±0.53b(a)
HTNE-2^{CT}	C	T	21	155.50±5.38a(a)	149.20±3.12a(a)	54.87±1.61a(a)	55.23±1.86a(a)	59.17±2.07a(a)	60.79±3.24a(a)

Note: HTNE-1, HTNE-2 and BC_1F_{2-3} grown in open field and net-room with 60 and 120 kg-N/ha application. HTNE-1 (♀), Xindao34, mother line without SNPs in *OsNRT2.3*. HTNE-2 (♂), Ka821asq, father line which co-exist the SNPs before *OsNRT2.3* ATG 111 bp (T mutant to C), and 7 bp (G mutant to T). HTNE-1^{TG}, HTNE-1 from recombination, *OsNRT2.3* alleles same as HTNE-1. HTNE-2^{CT}, homozygotes at -111 and -7 bp before *OsNRT2.3*, *OsNRT2.3* alleles same as HTNE-1. The letters for statistical analysis in () indicate that there is significant difference at 0.05 level among open field and net room of the same line, and the letters for statistical analysis out () indicate that there is significant difference at 0.05 level among different plants under the same condition, open field or net room.

Table S2. Statistical analysis the protein amount of OsNRT2.3a and OsNRT2.3b

	OsNRT2.3a		OsNRT2.3b	
	25℃	32℃	25℃	32℃
HTNE-1	0.7±0.1a(a)	0.5±0.05b(b)	0.5±0.1a(a)	0.6±0.005a(a)
HTNE-2	0.9±0.05a(a)	1.0±0.1a(a)	0.8±0.2b(b)	1.7±0.1a(a)
WT	0.6±0.07b(a)	0.5±0.04a(b)	0.6±0.1b(a)	0.6±0.02b(a)
Tilling F_{2-3}(aa）	1.3±0.3a(a)	0.4±0.05a(b)	1.0±0.05a(b)	1.7±0.1a(a)
Nip	1.4±0.1a(a)	1.1±0.1a(b)	0.9±0.01a(a)	0.8±0.05a(a)
OE1	0.9±0.05b(a)	0.8±0.01a(a)	0.6±0.01b(a)	0.6±0.02b(a)
OE2	1.0±0.05b(a)	0.8±0.1a(a)	0.7±0.02b(a)	0.5±0.02b(a)
Nip	1.4±0.1b(a)	1.1±0.1b(b)	0.9±0.01b(a)	0.8±0.05b(a)
negative control	1.45±0.1b(a)	1.3±0.05b(a)	0.8±0.05b(a)	0.8±0.05b(a)
mATG	2.1±0.07a(a)	1.5±0.1a(b)	1.6±0.1a(a)	1.6±0.2a(a)

Note: HTNE-1, lines without SNPs in *OsNRT2.3*. HTNE-2, plants which co-exist the SNPs upstream *OsNRT2.3* ATG 111 bp (T mutant to C), and 7 bp (G mutant to T). The protein amount of tilling F_{2-3} (aa) was taken from T11 F_{2-3} (aa) and T12 F_{2-3} (aa). The protein amount of OE1 and OE2 was taken from over-expression sNRT2.3-1 and sNRT2.3-2 lines, respectively. The protein amount of negative control and mATG was taken from aATG, bATG and mATG lines. The letters in () indicate that there is significant difference at 0.05 level among different temperature of the same line, and the letters out () indicate that there is significant difference at 0.05 level among different plants under the same condition, 25℃ or 32℃.

Table S3. Primers sequences used in this study

Primer	Sequence
p1505bp-F	ATATACCGTGAGATTTGATGC
p1505bp-R	CTCCAACACGTGGTAGCAAGGC
OsNRT2.3-F	ATGGAGGCTAAGCCGGTGGC
OsNRT2.3-R	CACCCCGGCCGGCGACGCGT
Ribo-*OsActin*-F	CCTCGTCTCGACCTTGCTGGG
Ribo-*OsActin*-R	GAGAACAAGCAGGAGGACGGC
Ribo-*OsNRT2.3a*-F	GCTCATCCGCGACACCCT
Ribo-*OsNRT2.3a*-R	GTCGAAGCGGTCGTAGAA
Ribo-*OsNRT2.3b*-F	CGTTCGCCGTGTT
Ribo-*OsNRT2.3b*-R	TCGAAGCGGTCGTAGAAG
sNRT2.3-1	ACGUGUUGGAGAUGGAGGCUAAGC
sNRT2.3-2	AGGCGACGGAGUUCUGGCUCUUCU
MIMIC-IPS1-F	CGCGGATCCAAGAAAAATGGCCATCCCCTAGC
MIMIC-IPS1-R	GCGGAGCTCAAGAGGAATTCACTATAAAGAG
sNRT2.3-1-bATG-F	GCTTAGCCTCTACCATCTCCAACACGTAGCTTCGGTTCC
sNRT2.3-1-bATG-R	ACGTGTTGGAGATTAGGGAGGCTAAGCTTTCTAGAGG
sNRT2.3-1-aATG-F	GCTTAGCCTCCATCTACTCCAACACGTAGCTTCGGTTCCCCTCGGAATCA
sNRT2.3-1-aATG-R	ACGTGTTGGAGATTAGGGAGGCTAAGCTTTCTAGAGGGAGATAAACAAAA
sNRT2.3-1-mATG-F	GCTTAGCCTCCCTAATCTCCAACACGTAGCTTCGGTTCCCCTCGGAATCA
sNRT2.3-1-mATG-R	ACGTGTTGGAGATTAGGGAGGCTAAGCTTTCTAGAGGGAGATAAACAAAA

Table S4. Primers sequences for Target-specific DMS-MaPseq

For primer extension assay	Sequence
18SrRNA RT Rev	AACTGATTTAATGAGCCATTCGCAG
**For target specific DMS-MaPseq**	
OsNRT2.3a-F	AGTCACTAGCTAAGCTGCTA
OsNRT2.3a-RT-R	GCCGGTGTGGTGAGGAGGATCAG
OsNRT2.3b-F	TCCCACCGGTCGCGTAAGAT
OsNRT2.3b-RT-R	CGAACACGGCGAACGTGGACAC
OsNRT1.1B-F	CGTCAGCATGACATACGCAAG
OsNRT1.1B-RT-R	CCTCCTGCCCTGCACACTATCAGTATAAAAGACG
